# Supplementary material for: Trends in oxygenate/hydrocarbon selectivity for electrochemical CO(2) reduction to C2 products
Source: Nat Commun. 2022 Mar 17;13:1399. doi: 10.1038/s41467-022-29140-8 (PMC8931056; doi:10.1038/s41467-022-29140-8)
Supplement: Supplementary file 1 — Supplementary Information [file 41467_2022_29140_MOESM1_ESM.pdf]

# 1. Supplementary Information for: Trends in Oxygenate/Hydrocarbon Selectivity for Electrochemical $CO_{(2)}$ Reduction to $C_2$ Products

## Supplementary Note 1: General calculation details for the adsorption energies

Reaction energetics were calculated with density functional theory (DFT) with a periodic plane-wave implementation and ultrasoft pseudopotentials using the QUANTUM ESPRESSO code,[1] interfaced with the Atomistic Simulation Environment (ASE).[2] We applied the BEEF-vdW functional, which provides a reasonable description of van der Waals forces while maintaining an accurate prediction of chemisorption energies.[3] Plane-wave and density cutoffs were 500 and 5000 eV, respectively, with a Fermi-level smearing width of 0.1 eV. psLib ultrasoft pseudopotentials were chosen. The adsorption energies on (100) surfaces of *fcc* transition metals were evaluated using four-layer (3 × 3) supercells with the bottom two layers constrained and a vacuum layer of 20 Å, and [4 × 4 × 1] Monkhorst-Pack **k**-point grids[4] were used.

All the cell sizes and corresponding Monkhorst-Pack **k**-point grids for other Cu facets and intermetallic surfaces could be found in **Supplementary Table 1**.

**Supplementary Table 1. Computational details for materials and surfaces shown in the ($\Delta G_{C*}$, $\Delta G_{OH*}$) selectivity maps.**

| Materials | Surfaces | Unit cell size | Monkhorst-Pack **k**-point grids |
|---|---|---|---|
| Various Cu | (110) | (2 × 3) | [4 × 4 × 1] |
| | (211) | (1 × 3) | [4 × 4 × 1] |
| | (511) | (3 × 1) | [4 × 4 × 1] |
| | (310) | (3 × 1) | [3 × 6 × 1] |
| $L1_2$ intermetallics | $A_3B$ (100) with AB surface termination | (2√2 × 2√2) | [4 × 4 × 1] |
| $L1_0$ intermetallics | AB (100) with AB surface termination | (2√2 × 2√2) | [4 × 4 × 1] |
| Ni-Ga | $Ni_5Ga_3$ (111) | (2 × 1) | [4 × 4 × 1] |

All the surfaces have four layers with the top two layers relaxed. (100) surface orientations were selected due to the uniqueness of four-fold hollow site in stabilizing the C* intermediate compared to corresponding three-fold hollow site. The role of four-fold hollow sites in enabling selective $C_2$ formation through $CO_{(2)}R$ has been elucidated in our previous work.[5] The AB-type surface terminations for $L1_2$ and $L1_0$ intermetallics were selected due to the relatively low surface energy.[5]

All structures were optimized until the force components were less than 0.05 eV Å$^{-1}$. A dipole correction was applied to decouple the electrostatic interaction between the periodically repeated slabs. All relaxed structures and energetics are available on the Catalysis-Hub database.[6] All computational data linked to the list of corresponding electronic energies has been released as part of the Catalysis-hub.org repository[6] under https://www.catalysis-hub.org/publications/PengTrends2022.

## Supplementary Note 2: Gibbs free energy correction

To directly obtain the reaction energies $\Delta G_{rxn}$ from energetic scaling relationship and use them for the selectivity maps, we used adsorption free energies, $\Delta G$, instead of electronic energies to construct those scaling relationships. Thus, $\Delta G$ is affected by the selected gas-phase free energies as the references. Here we made the same assumption as Peterson *et al.* did in 2010[7] that gaseous products in the pathway were calculated at partial pressures corresponding to the Faradaic yields reported by Hori et al,[8] and liquid (or dissolved) products in the pathway were calculated at a molarity of 1 mol $L^{-1}$ (M). The fugacities we used for gaseous products are the same as in Ref.[7]. Acetylene ($C_2H_2$), ethane ($C_2H_6$), ethenone ($CH_2CO$), acetic acid ($CH_3COOH$), acetaldehyde ($CH_3CHO$), and vinyl alcohol ($CH_2CHOH$) are not counted for Faradaic yields in Ref.[8]. We then assumed that all stable $C_2$ HC (i.e. $C_2H_2$ and $C_2H_6$) have the same fugacity as ethylene ($C_2H_4$), all stable $C_2$ Oxy except ethanol ($CH_3CH_2OH$), i.e. $CH_3COOH$ and $CH_3CHO$, have the fugacities calculated based on Henry's law, and the two unstable molecules of $CH_2CO$ and $CH_2CHOH$ have a very low fugacity of 0.1 Pa. The constants applied in Henry's law, for $CH_3COOH$ and $CH_3CHO$, are $1.82\times10^{-4}$ and $6.67\times10^{-2}$ bar $M^{-1}$,[9] corresponding to fugacities of 18.2 and 6670 Pa for $CH_3COOH$ and $CH_3CHO$, respectively. Therefore, the contribution to the chemical potential $\mu$ of gas-phase or dissolved molecules could be found in **Supplementary Table 2**. Note that a DFT correction of +0.15 eV was added for gas-phase molecules containing C=O double bonds ($CH_2CO$, $CH_3COOH$, and $CH_3CHO$), according to Christensen *et al.*[10], and a DFT correction of +0.09 eV was added for hydrogen gas ($H_2$) due to the systematic error caused by the BEEF-vdW functional, in accordance with Studt *et al.*[11]

**Supplementary Table 2. Molecular free energy correction.** Assumed fugacity for each non-adsorbate species, along with calculated electronic energies ($E_{raw}$), DFT correction ($E_{BEEF\text{-}vdW}$), corrected electronic energies ($E_{ele} = E_{raw} + E_{BEEF\text{-}vdW}$), zero point energies (ZPE), enthalpic temperature correction, entropy contribution ($-TS$), chemical potential ($\mu = E_{ele} + \text{ZPE} + \int C_p dT - TS$), and the total correction ($\mu - E_{ele} = \text{ZPE} + \int C_p dT - TS$). $H_2$ is the value used for gaseous hydrogen, $H_2$ (ref) is used for the computational hydrogen electrode.

| Species | Fugacity (Pa) | $E_{raw}$ (eV) | $E_{BEEF\text{-}vdW}$ (eV) | $E_{ele}$ (eV) | ZPE (eV) | $\int C_p dT$ (eV) | $-TS$ (eV) | $\mu$ (eV) | $\mu - E_{ele}$ (eV) |
|---|---|---|---|---|---|---|---|---|---|
| *Used for energetic analyses and microkinetic modeling* | | | | | | | | | |
| CO(g) | 5562 | 0.00 | | 0.00 | 0.13 | 0.09 | −0.69 | −0.46 | −0.46 |
| $H_2$(g) | 30296 | −0.09 | 0.09 | 0.00 | 0.27 | 0.09 | −0.43 | −0.07 | −0.07 |
| $CH_4$(g) | 20467 | −2.80 | | −2.80 | 1.20 | 0.10 | −0.62 | −2.10 | 0.69 |
| $C_2H_4$(g) | 13942 | −3.14 | | −3.14 | 1.37 | 0.11 | −0.73 | −2.39 | 0.75 |
| $C_2H_2$(g) | 13942 | −1.00 | | −1.00 | 0.72 | 0.10 | −0.67 | −0.85 | 0.15 |
| $C_2H_6$(g) | 13942 | −4.76 | | −4.76 | 2.00 | 0.12 | −0.76 | −3.39 | 1.36 |
| $CH_2CO$(g) | 0.1 | −1.54 | 0.15 | −1.39 | 0.85 | 0.12 | −1.11 | −1.53 | −0.14 |
| $CH_3COOH$(g) | 18.2 | −2.99 | 0.15 | −2.84 | 1.64 | 0.15 | −1.13 | −2.17 | 0.67 |
| $CH_3CHO$(g) | 6670 | −2.87 | 0.15 | −2.72 | 1.48 | 0.14 | −0.89 | −2.00 | 0.72 |
| $CH_2CHOH$(g) | 0.1 | −2.35 | | −2.35 | 1.50 | 0.13 | −1.16 | −1.87 | 0.48 |
| $CH_3CH_2OH$(g) | 3196 | −3.69 | | −3.69 | 2.13 | 0.15 | −0.93 | −2.34 | 1.35 |

| | | | | | | | | | |
|---|---|---|---|---|---|---|---|---|---|
| $H_2O$(g) | 3534 | 0.00 | | 0.00 | 0.57 | 0.10 | −0.67 | 0.00 | 0.00 |
| $H_2$(g) (ref) | 101325 | −0.09 | 0.09 | 0.00 | 0.27 | 0.09 | −0.40 | −0.04 | −0.04 |
| *Used only for calculating equilibrium potentials in Table S3* | | | | | | | | | |
| CO(g) | 101235 | 0.00 | | 0.00 | 0.13 | 0.09 | −0.61 | −0.39 | −0.39 |
| $H_2$(g) | 101235 | −0.09 | 0.09 | 0.00 | 0.27 | 0.09 | −0.40 | −0.04 | −0.04 |
| $CH_4$(g) | 101235 | −2.80 | | −2.80 | 1.20 | 0.10 | −0.57 | −2.06 | 0.73 |
| $C_2H_4$(g) | 101235 | −3.14 | | −3.14 | 1.37 | 0.11 | −0.68 | −2.34 | 0.80 |
| $C_2H_2$(g) | 101235 | −1.00 | | −1.00 | 0.72 | 0.10 | −0.62 | −0.80 | 0.20 |
| $C_2H_6$(g) | 101235 | −4.76 | | −4.76 | 2.00 | 0.12 | −0.71 | −3.34 | 1.42 |
| $CH_2CO$(g) | 101235 | −1.54 | 0.15 | −1.39 | 0.85 | 0.12 | −0.75 | −1.18 | 0.22 |
| $CH_3COOH$(g) | 101325 | −2.99 | 0.15 | −2.84 | 1.64 | 0.15 | −0.90 | −1.95 | 0.89 |
| $CH_3COOH$ (aq, 1 M)[a] | | | | | | | | −2.17 | |
| $CH_3CHO$(g) | 101325 | −2.87 | 0.15 | −2.72 | 1.48 | 0.14 | −0.82 | −1.93 | 0.79 |
| $CH_3CHO$ (aq, 1 M)[a] | | | | | | | | −2.00 | |
| $CH_2CHOH$(g) | 101235 | −2.35 | | −2.35 | 1.50 | 0.13 | −0.80 | −1.52 | 0.83 |
| $CH_3CH_2OH$(g) | 101325 | −3.69 | | −3.69 | 2.13 | 0.15 | −0.84 | −2.25 | 1.44 |
| $CH_3CH_2OH$ (aq, 1 M)[a] | | | | | | | | −2.39 | |
| $H_2O$(g) | 3534 | 0.00 | | 0.00 | 0.57 | 0.10 | 0.00 | 0.09 | 0.00 |

a. For the 1 M liquid products such as $CH_3COOH$, $CH_3CHO$, and $CH_3CH_2OH$ dissolving in aqueous solution, the free energies were calculated from those of gaseous products at 101325 Pa using the Henry's law. The Henrys-law constants for $CH_3COOH$, $CH_3CHO$, and $CH_3CH_2OH$ are $1.82\times10^{-4}$, $6.67\times10^{-2}$, and $4.55\times10^{-3}$ bar $M^{-1}$, respectively.[9]

$E_{ele}$ and $\mu$ are referenced to the gas-phase electronic energy of CO, $H_2O$, and $H_2$ (ref) and $\mu$ is different from the free energy change ($\Delta G$, referenced to the gas-phase free energy) shown in **Figures 2, 5, Supplementary Figures 1, 4–6, 8–10, 15, 16** and **Supplementary Table 12**. For example, $\mu_{CH4} = E_{ele\_CH4} + (ZPE + \int C_p dT - TS)_{CH4}$ while $\Delta G_{CH4} = \mu_{CH4} - (\mu_{CO} + 2 \times \mu_{H2\,(ref)} - \mu_{H2O})$.

To validate the afore obtained gas-phase energies, the energies at the standard condition (298 K, 101325 Pa) are used to calculate the equilibrium potentials for comparison with the experimental values obtained from the NIST Chemistry Webbook (https://webbook.nist.gov/chemistry/) and Langes Handbook of Chemistry. Most of the experimental data has been collected in the Supporting Information of Ref.[9] The standard reaction enthalpies ($\Delta H^0$) and equilibrium potentials ($U_{eq}$) are compared in **Supplementary Table 3**. It is notable that the errors in $\Delta H^0$ and $U_{eq}$ are within ±5 kJ $mol^{-1}$ and ±0.02 V, respectively. Ethenone ($CH_2CO$) is an exception herein and the errors in $\Delta H^0$ are calculated as 18.65 and −20.55 kJ $mol^{-1}$, depending on the experimental data from different sources. The experimentally measured $\Delta H^0$ varies by ~40 kJ $mol^{-1}$. Overall, these results indicate that with the above DFT corrections applied to $H_2$, $CH_2CO$, $CH_3COOH$, and $CH_3CHO$, reasonable agreement with thermochemical data is achieved.

**Supplementary Table 3. Comparison between theoretically calculated energies with thermochemical data.** Considering that the reaction mechanism is mainly discussed in this work using CO as the carbon source, all the reactions could be in general written as $x$ CO + $n$ ($H^+ + e^-$) → product + $y$ $H_2O$. Note that $H_2O$ herein is gaseous $H_2O$ with a fugacity of 3534 Pa at 298 K (a condition we used in this work), which is in equilibrium with liquid $H_2O$ at the standard condition.

| Products | Experimental data[a] | | Computational data | | | Error | |
|---|---|---|---|---|---|---|---|
| | $\Delta H^0$ (kJ mol$^{-1}$) | $U_{eq}$ (V vs. RHE) | $\Delta H^0$ (eV) | $\Delta H^0$ (kJ mol$^{-1}$) | $U_{eq}$ (V vs. RHE) | $\Delta H^0$ (kJ mol$^{-1}$) | $U_{eq}$ (V) |
| $CH_4$ | −206.2 | 0.26 | −2.13 | −205.7 | 0.25 | 0.51 | −0.01 |
| $C_2H_4$ | −210.2 | 0.17 | −2.22 | −213.7 | 0.16 | −3.55 | −0.01 |
| $C_2H_2$ | −35.2 | | −0.37 | −35.6 | −0.04 | −0.43 | |
| $C_2H_6$ | −346.6 | 0.24 | −3.56 | −343.1 | 0.22 | 3.55 | −0.02 |
| $CH_2CO$ | −108.0[b] (−68.8)[b] | | −1.08 | −89.3 | 0.18 | 18.65 (−20.55) | |
| $CH_3COOH$ | −212.0 | 0.32 | −2.22 | −214.3 | 0.33 | −2.30 | 0.01 |
| $CH_3CHO$ | −186.9 | 0.17 | −1.97 | −190.2 | 0.17 | −3.32 | 0.00 |
| $CH_2CHOH$ | −148.8 | | −1.58 | −152.7 | 0.15 | −3.92 | |
| $CH_3CH_2OH$ | −255.6 | 0.19 | −2.64 | −255.1 | 0.17 | 0.50 | −0.02 |

a. The experimental data was directly adopted from Ref.[9] .

Similarly, the free energy correction to the adsorbates could be found in **Supplementary Table 4**. Modes of vibration were found by performing a normal-mode analysis; all vibrations were treated in the harmonic oscillator approximation. All vibrations used for free energy correction were obtained on Cu(100).

**Supplementary Table 4. Adsorbate free energy correction.** Contributions to the adsorbate free energy from the ZPE correction, enthalpic temperature correction, entropy, and the total free energy correction, respectively.

| Adsorbates | ZPE (eV) | $\int C_p dT$ (eV) | $-TS$ (eV) | $\mu - E_{ele}$ (eV) | Adsorbates | ZPE (eV) | $\int C_p dT$ (eV) | $-TS$ (eV) | $\mu - E_{ele}$ (eV) |
|---|---|---|---|---|---|---|---|---|---|
| CO* | 0.17 | 0.07 | −0.16 | 0.09 | $CH_2COH$* | 1.18 | 0.09 | −0.16 | 1.11 |
| COH* | 0.46 | 0.09 | −0.17 | 0.37 | $OCH_2CH$* | 1.19 | 0.10 | −0.17 | 1.13 |
| C* | 0.09 | 0.02 | −0.03 | 0.08 | CHCHOH* | 1.22 | 0.08 | −0.16 | 1.15 |
| CH* | 0.34 | 0.03 | −0.05 | 0.33 | CCH* | 0.44 | 0.09 | −0.16 | 0.37 |
| $CH_2$* | 0.59 | 0.06 | −0.10 | 0.55 | $OCHCH_3$* | 1.50 | 0.09 | −0.20 | 1.38 |
| $CH_3$* | 0.91 | 0.08 | −0.15 | 0.84 | $CH_3COH$* | 1.46 | 0.11 | −0.24 | 1.33 |
| OCCO* | 0.42 | 0.10 | −0.18 | 0.35 | $OCH_2CH_2$* | 1.49 | 0.10 | −0.19 | 1.40 |
| OCCOH* | 0.72 | 0.11 | −0.19 | 0.65 | $CH_2CHOH$* | 1.51 | 0.07 | −0.17 | 1.42 |
| CCO* | 0.33 | 0.09 | −0.15 | 0.27 | $CCH_2$* | 0.74 | 0.08 | −0.16 | 0.66 |
| CHCO* | 0.60 | 0.09 | −0.22 | 0.47 | CHCH* | 0.75 | 0.08 | −0.13 | 0.69 |
| CCHO* | 0.57 | 0.06 | −0.11 | 0.53 | $OCH_2CH_3$* | 1.84 | 0.10 | −0.19 | 1.75 |
| CCOH* | 0.56 | 0.08 | −0.18 | 0.46 | $CCH_3$* | 1.07 | 0.05 | −0.09 | 1.03 |
| $CH_2CO$* | 0.90 | 0.10 | −0.17 | 0.83 | $CHCH_2$* | 1.09 | 0.09 | −0.18 | 1.01 |
| OCHCH* | 0.91 | 0.10 | −0.20 | 0.81 | $CHCH_3$* | 1.34 | 0.10 | −0.20 | 1.23 |

| | | | | | | | | |
|---|---|---|---|---|---|---|---|---|
| $CHCOH^*$ | 0.89 | 0.11 | −0.21 | 0.79 | $CH_2CH_3^*$ | 1.68 | 0.08 | −0.15 | 1.61 |
| $CH_3CO^*$ | 1.20 | 0.09 | −0.17 | 1.11 | $H^*$ | 0.13 | 0.01 | −0.02 | 0.12 |
| $OCHCH_2^*$ | 1.20 | 0.10 | −0.21 | 1.10 | $OH^*$ | 0.32 | 0.07 | −0.15 | 0.24 |

## Supplementary Note 3: Solvation correction

According to previous analysis,[12, 13] the solvation corrections ($E_{\mathrm{solv\ corr}}$) to $CO^*$, $COH^*$, $OCCO^*$, $OCCOH^*$, $CCO^*$, and $CHCO^*$ are summarized in **Supplementary Table 5**. The binding energy after solvation correction $\Delta E_{\mathrm{solv}} = \Delta E_{\mathrm{vacuum}} - E_{\mathrm{solv\ corr}}$, where $\Delta E_{\mathrm{vacuum}}$ is the adsorption energy calculated without explicit solvents (in vacuum), $E_{\mathrm{solv\ corr}}$ is the energetic correction listed in **Supplementary Table 5**, and $\Delta E_{\mathrm{solv}}$ is the solvation-corrected adsorption energy used in this work. Three-layer (4 × 3) supercells with a monolayer of explicit water, *i.e.* the same setting for electrochemical barrier calculations, were employed to investigate the solvation effect on several key intermediates identified in this work, including $CH_2CO^*$, $OCHCH^*$, $CHCOH^*$, $CHCHOH^*$, $CCH^*$, $CH_2CHOH^*$, and $OCHCH_3^*$. Except $CH_2CHOH^*$ and $OCHCH_3^*$, the studied five important $C_2$ intermediates have a similar $E_{\mathrm{solv\ corr}}$ of around −0.10 eV, while $CH_2CHOH^*$ and $OCHCH_3^*$ have a distinct $E_{\mathrm{solv\ corr}}$ of −0.51 eV and −0.25 eV, respectively. Due to the similar binding geometry, $OCH_2CH_3^*$ was assumed to have the same $E_{\mathrm{solv\ corr}}$ of −0.25 eV as $OCHCH_3^*$. The rest of $C_2$ species were assumed to have a $E_{\mathrm{solv\ corr}}$ of −0.10 eV according to the results obtained for $CH_2CO^*$, $OCHCH^*$, $CHCOH^*$, $CHCHOH^*$, and $CCH^*$. Further works employing other methods such as implicit solvents[14, 15] and *ab initio* molecular dynamics[13] to determine the solvation effect would be future directions.

**Supplementary Table 5. Solvation corrections.**

| Adsorbates | $E_{\mathrm{solv\ corr}}$ (eV) | Adsorbates | $E_{\mathrm{solv\ corr}}$ (eV) |
|---|---|---|---|
| $CO^*$[13] | −0.06 | $OCCO^*$[13] | −0.25 |
| $COH^*$[13] | −0.11 | $OCCOH^*$ | −0.30 |
| $C^*$ | 0 | $CCO^*$[12] | −0.10 |
| $CH^*$ | 0 | $CHCO^*$[12] | −0.10 |
| $CH_2^*$ | 0 | $CH_2CO^*$ | −0.10 |
| $CH_3^*$ | 0 | $OCHCH^*$ | −0.10 |
| $H^*$ | 0 | $CHCOH^*$ | −0.10 |
| $OH^*$ | 0 | $CH_2CHOH^*$ | −0.51 |
| $CO_{HP}$ | 0 | $OCH_{(2)}CH_3^*$ | −0.25 |
| | | The rest of $C_2$ species | −0.10 |

HP: Helmholtz plane

## Supplementary Note 4: Interface electric field model and field correction

Cation-induced fields lead to dramatic stabilizations of the $C_2$ species involved.[16] Similar to previous works,[5, 12] we consider the field effect on the energetics as follows. We applied a sawtooth potential in the *z*-direction for structures of the adsorbates in vacuum. The interaction energy between the adsorbate and the interfacial field can be described by

$$\Delta\Delta E = \mu\varepsilon - \frac{1}{2}\alpha\varepsilon^2 + \ldots \quad (1)$$

where $\Delta\Delta E$ is the change in binding energy $\Delta E$, $\varepsilon$ is the electric field strength, and $\mu$ and $\alpha$ are the intrinsic dipole moment and polarizability of the adsorbate, respectively.[17] The parameters $\mu$ and $\alpha$ of the key intermediates are shown in **Supplementary Table 6**. The parameterization was performed on a (3×3×4) Cu(100) slab.

**Supplementary Table 6. Parameters that describe the field effect on adsorption energies.**

| $C_1$ species | $CO_{HP}$ | CO* | COH* | C* | CH* | |
|---|---|---|---|---|---|---|
| $\mu$ ($e$Å) | 0.0357 | 0.0440 | −0.125 | 0.135 | −0.00870 | |
| $\alpha$ ($e$Å$^2$ V$^{-1}$) | 0.254 | 0.302 | 0.514 | −0.0752 | 0.0340 | |
| $C_2$ species ($n \leq 2$) | OCCO* | OCCOH* | TS: C-CO | CCO* | | |
| $\mu$ ($e$Å) | 0.672 | 0.239 | 0.225 | 0.357 | | |
| $\alpha$ ($e$Å$^2$ V$^{-1}$) | 0.466 | 0.652 | 0.612 | 0.530 | | |
| $C_2$ species ($n$ = 3, 4) | CHCO* | CCHO* | CCOH* | $CH_2CO$* | OCHCH* | CHCOH* |
| $\mu$ ($e$Å) | 0.127 | 0.498 | −0.341 | 0.099 | 0.017 | −0.219 |
| $\alpha$ ($e$Å$^2$ V$^{-1}$) | 0.540 | 0.438 | 0.477 | 0.434 | 0.648 | 1.011 |
| $C_2$ species ($n$ = 5) | $CH_3CO$* | $OCHCH_2$* | $CH_2COH$* | $OCH_2CH$* | CHCHOH* | CCH* |
| $\mu$ ($e$Å) | −0.199 | −0.0709 | −0.326 | −0.0204 | −0.263 | −0.180 |
| $\alpha$ ($e$Å$^2$ V$^{-1}$) | 0.593 | 0.592 | 0.518 | 0.688 | 0.743 | 0.477 |
| $C_2$ species ($n$ = 6) | $OCHCH_3$* | $CH_3COH$* | $OCH_2CH_2$* | $CH_2CHOH$* | $CCH_2$* | CHCH* |
| $\mu$ ($e$Å) | −0.998 | −0.641 | −0.0446 | −0.223 | −0.185 | −0.174 |
| $\alpha$ ($e$Å$^2$ V$^{-1}$) | 1.088 | 0.817 | 0.708 | 0.945 | 0.781 | 0.432 |
| $C_2$ species ($n \geq 7$) | $OCH_2CH_3$* | $CCH_3$* | $CHCH_2$* | $CHCH_3$* | $CH_2CH_3$* | |
| $\mu$ ($e$Å) | −0.157 | −0.134 | −0.118 | −0.105 | −0.127 | |
| $\alpha$ ($e$Å$^2$ V$^{-1}$) | 0.634 | 0.667 | 1.010 | 0.916 | 0.943 | |

Note: $CO_{HP}$: CO in the Helmholtz plane; CH* is insensitive to the field and thus not considered for the field correction; parameters for CCO* are different from those reported in our previous work, in which a (4 × 3) supercell of Cu(100) was employed for parameterization. In this work, the setting was corrected to be the same as for all other species. $n$ is the proton-electron transfer (PET) number relative to CO.

The field dependence of key intermediates is shown in **Supplementary Figure 1** using the above parameters of $\mu$ and $\alpha$ and Eq. (1). In general, oxygenated surface species, especially those featured by a carbonyl group (*e.g.* CCO*, CHCO*, $OCHCH_3$*), are more sensitive to the interfacial field because of the stronger polarity of these species than hydroxylated or hydrocarbon species. Also, with the more hydrogen contained in the molecular backbone, the surface specie normally exhibited weaker sensitivity to the interfacial field. For instance, species shown in **Supplementary Figure 1a** exhibit the most obvious field dependency.

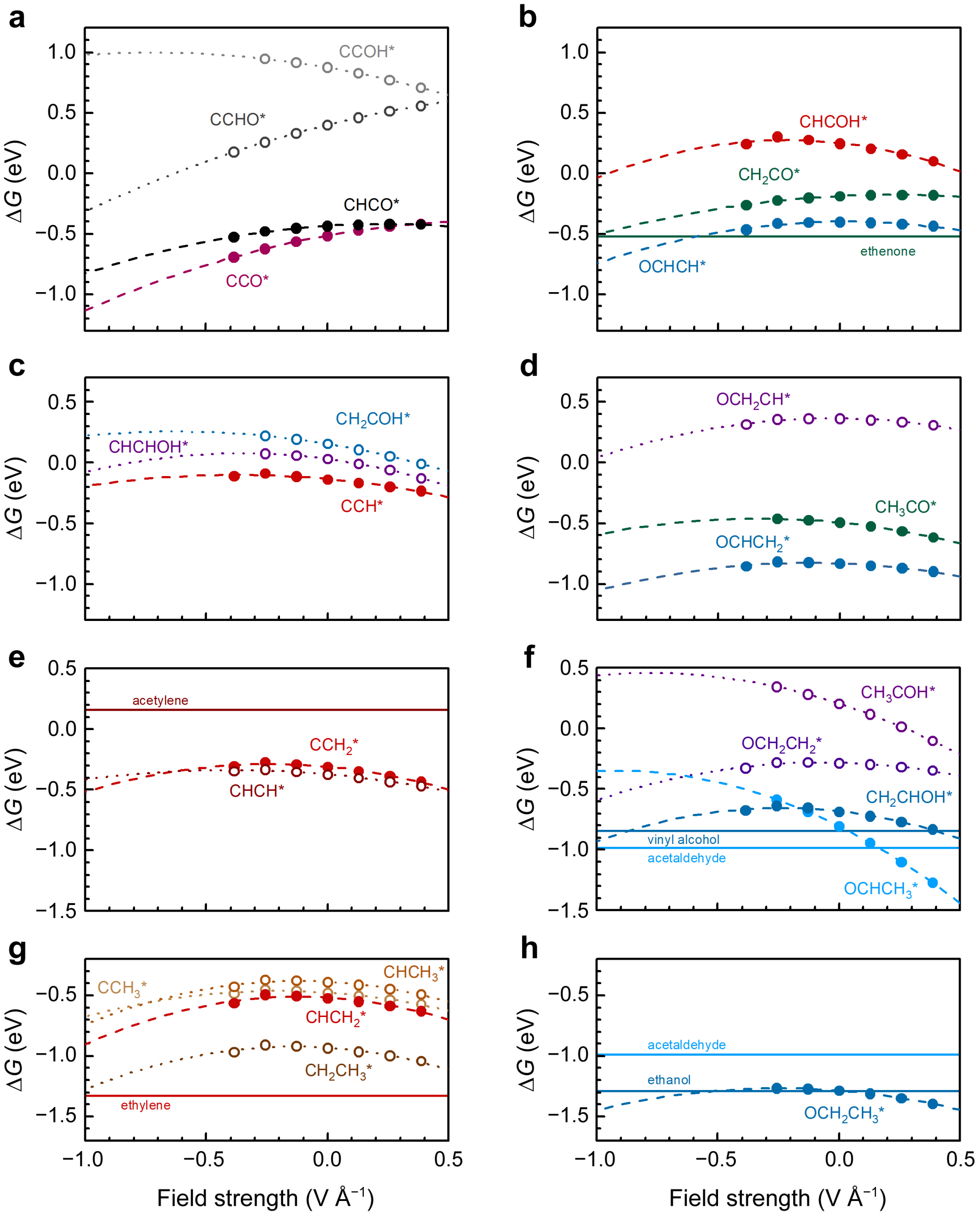


**Supplementary Figure 1. Field effect on energetics of key intermediates.** Gibbs adsorption free energies of key $C_2$ intermediates under various field strengths, classified by (**a**) $n = 2, 3$, (**b**) $n = 4$, (**c, d**) $n = 5$, (**e, f**) $n = 6$, and (**g, h**) $n \geq 7$. In subfigures **c**–**h**, the left panel shows energies associated mainly with the CHCOH pathway, while the right panel shows those associated with the OCHCH/$CH_2CO$ pathway. Kinetically more accessible intermediates refer to solid circles and dashed lines, while those less accessible intermediates refer to hollow circles and dotted lines. Solid horizontal lines in **b** and **e**–**h** represent the desorbed molecular species in gas or solution phase. All $\Delta G$ are referenced to Gibbs free energies of CO(g), $H_2$(g), and $H_2O$(g).

Then, we correlate the interfacial electric field strength to the applied potential for the Cu(100) electrode. The detailed method was discussed in prior works.[5, 18] According to the classical double layer theory,[19] the electric field strength $\varepsilon$ is derived from

$$\varepsilon = \frac{\Phi_{\mathrm{M}} - \Phi_{\mathrm{M,\,PZC}}}{d} \tag{2}$$

where $\Phi_{\mathrm{M}}$ and $\Phi_{\mathrm{M,\,PZC}}$ are applied potential of a metal electrode and the zero-charge potential of this metal electrode, respectively, and $d$ is the distance between the positively (*i.e.* the center of the Helmholtz plane) and negatively charged planes. $\Phi_{\mathrm{M,\,PZC}}$ approximates the electrolyte potential at the Helmholtz plane ($\Phi_{\mathrm{HP}}$), and the potential difference $\Phi_{\mathrm{M}} - \Phi_{\mathrm{HP}}$ is defined as the driving force for the interfacial charge transfer that builds the electric field.[20] Eq. (2) explicitly introduces the potential dependence of $\varepsilon$. Herein experimentally measured $\Phi_{\mathrm{M,\,PZC}} = -0.54$ V vs. the standard hydrogen electrode (SHE) for Cu(100)[18, 21] and $d$ = 1.2 Å were employed in our simulation. Sensitivity analysis on $d$ was performed previously.[18]

With including the above $U_{\mathrm{SHE}}$-dependent field effect and the computational hydrogen electrode (CHE) model[22] that brings in energetic correction to electrons at a $U_{\mathrm{RHE}}$ scale, the electronic adsorption energy of a certain specie at a given $U_{\mathrm{SHE/RHE}}$ is given by

$$\Delta E_{\mathrm{ads}}^{U_{\mathrm{SHE/RHE}}} = \underbrace{\Delta E_{\mathrm{ads}}^{U_{\mathrm{RHE}}=0} + neU_{\mathrm{RHE}}}_{(U_{\mathrm{RHE}}\ \text{scale})} + \underbrace{\frac{\mu}{d}(U_{\mathrm{SHE}} - U_{\mathrm{M,\,PZC}}) - \frac{1}{2}\frac{\alpha}{d^2}(U_{\mathrm{SHE}} - U_{\mathrm{M,\,PZC}})^2}_{(U_{\mathrm{SHE}}\ \text{scale})} \tag{3}$$

where $\Delta E_{\mathrm{ads}}^{U_{\mathrm{RHE}}=0}$ is the adsorption energy at $U_{\mathrm{RHE}}$ = 0 V (referenced to gas-phase CO, $H_2O$, and $H_2$), $n$ is the number of transferred electron, $U_{\mathrm{M,\,PZC}} = -0.54$ V vs. SHE for Cu(100), while $U_{\mathrm{SHE}}$ and $U_{\mathrm{RHE}}$ are correlated through the pH of the bulk electrolyte

$$U_{\mathrm{RHE}} = U_{\mathrm{SHE}} + 0.059\mathrm{pH} \tag{4}$$

Note that the $U_{\mathrm{SHE}}$-scale term in Eq. (3) is only applied to field-sensitive species as shown in **Supplementary Figure 1** and other species follow the $U_{\mathrm{RHE}}$-scale dependency. Lastly, we added the solvation corrections as described above to obtain the adsorption energies. The obtained adsorption energies used for microkinetic modeling are shown in **Supplementary Table 7**.

**Supplementary Table 7. Electronic energies $E_{\mathrm{ads}}^{U_{\mathrm{RHE}}=0}$ and chemical potentials $\mu_{\mathrm{ads}}^{U_{\mathrm{RHE}}=0}$ of species involved in the microkinetic modeling on Cu(100).**

| Conditions | vacuum | solv | | field correction applied? (*yes* or *no*) |
|---|---|---|---|---|
| Adsorbates | $E_{\mathrm{ads}}^{U_{\mathrm{RHE}}=0}$ (eV) | $E_{\mathrm{ads}}^{U_{\mathrm{RHE}}=0}$ (eV) | $\mu_{\mathrm{ads}}^{U_{\mathrm{RHE}}=0}$ (eV) | |
| CO* | −0.65 | −0.71 | −0.62 | *yes* |
| COH* | 0.34 | 0.23 | 0.60 | *yes* |
| C* | 0.38 | 0.38 | 0.46 | *yes* |
| CH* | −0.47 | −0.47 | −0.14 | *no* |
| $CH_2$* | −0.60 | −0.60 | −0.05 | *no* |
| $CH_3$* | −1.68 | −1.68 | −0.84 | *no* |
| OCCO* | −0.12 | −0.37 | −0.02 | *yes* |
| OCCOH* | −0.38 | −0.68 | −0.03 | *yes* |
| CCO* | −1.62 | −1.72 | −1.45 | *yes* |

| | | | | |
|---|---|---|---|---|
| $CHCO^*$ | −1.75 | −1.85 | −1.38 | *yes* |
| $CCHO^*$ | −0.98 | −1.08 | −0.55 | *yes* |
| $CCOH^*$ | −0.43 | −0.53 | −0.07 | *yes* |
| $CH_2CO^*$ | −1.89 | −1.99 | −1.16 | *yes* |
| $OCHCH^*$ | −2.11 | −2.21 | −1.40 | *yes* |
| $CHCOH^*$ | −1.40 | −1.50 | −0.71 | *yes* |
| $CH_3CO^*$ | −2.49 | −2.59 | −1.48 | *yes* |
| $OCHCH_2^*$ | −2.82 | −2.92 | −1.82 | *yes* |
| $CH_2COH^*$ | −1.84 | −1.94 | −0.83 | *yes* |
| $OCH_2CH^*$ | −1.66 | −1.76 | −0.63 | *yes* |
| $CHCHOH^*$ | −2.00 | −2.10 | −0.95 | *yes* |
| $CCH^*$ | −1.39 | −1.49 | −1.12 | *yes* |
| $OCHCH_3^*$ | −2.95 | −3.20 | −1.82 | *yes* |
| $CH_3COH^*$ | −2.01 | −2.11 | −0.78 | *yes* |
| $OCH_2CH_2^*$ | −2.61 | −2.71 | −1.31 | *yes* |
| $CH_2CHOH^*$ | −2.59 | −3.10 | −1.68 | *yes* |
| $CCH_2^*$ | −1.88 | −1.98 | −1.32 | *yes* |
| $CHCH^*$ | −1.99 | −2.09 | −1.40 | *yes* |
| $OCH_2CH_3^*$ | −3.82 | −4.07 | −2.32 | *yes* |
| $CCH_3^*$ | −2.45 | −2.55 | −1.52 | *yes* |
| $CHCH_2^*$ | −2.45 | −2.55 | −1.54 | *yes* |
| $CHCH_3^*$ | −2.58 | −2.68 | −1.45 | *yes* |
| $CH_2CH_3^*$ | −3.51 | −3.61 | −2.00 | *yes* |
| $H^*$ | 0.07 | 0.07 | 0.19 | *no* |
| $OH^*$ | −0.04 | −0.04 | 0.20 | *no* |
| $CO_{HP}$ | 0.00 | 0.00 | −0.45 | *yes* |

Note: $CO_{HP}$: CO in the Helmholtz plane

## Supplementary Note 5: Electrochemical barriers and hydronium vs. water as the hydrogen source for proton transfer

Electrochemical barriers of $C_2$ specie formation were calculated with (4 × 3) supercells and Monkhorst-Pack k-point grids of [3 × 4 × 1], respectively. All structures contained a three-layer transition metal slab, with atoms in the top layer relaxed and the rest fixed, along with a hydrogen-bonded water layer determined through minima hopping.[16] Minima hopping was used to determine the optimal water structure that interacts with the metal slabs without any intermediate adsorbed. Upon reaching the minimum structure, intermediates are added to conduct climbing-image nudged elastic band (CI-NEB) calculations to determine transition state (TS) geometries, with the forces on the climbing image converged to less than 0.05 eV $Å^{-1}$.[23] The spring constants were tightened for images close to the saddle point.[24] The plane wave and charge density cutoff, exchange-correlation functional, and other parameters were the same as those used for geometry optimizations. The charge extrapolation method[25, 26] was used to deduce the activation

barriers at constant potential.[27] The charge and work function for each state were calculated to estimate the energy change induced by charging. The charge was calculated through Bader analysis without including the transferring proton in the TS. The net dipole from the oriented water layer was found to be −1.3 eV,[28] and this value was subtracted from the calculated work function to correct for the net effect of using an oriented water layer in the simulations. A $H_3O^+$ ion was present in the initial state (IS) to act as the hydrogen source for protonation. Later on, we will introduce an approach to estimate alkaline PET barriers (water as the hydrogen source) from the barriers obtained under acidic conditions ($H_3O^+$). This approach was elucidated in details in our previous work.[5] The following section was adopted from the Supporting Information of Ref.[5] in case the approach could not be easily understood.

We note that all of the reported barriers are acidic barriers, *i.e.*, $H_3O^+$ as the hydrogen source. However, at neutral and alkaline conditions, the concentration of $H_3O^+$ is lower than $10^{-7}$ M in the aqueous solution at room temperature. Recent experimental and theoretical works also showed that water should be the dominant hydrogen source for PET in $CO_2$R and HER at neutral and alkaline conditions.[29, 30] However, currently it is not trivial to model alkaline barriers with water using DFT.[12, 30] Thus, we use hydronium as a proton source with an alkaline correction applied based on other works.[5, 12, 30-32] The following are detailed analysis regarding pH effects on the IS/FS energies and the choice of potential scales of RHE/SHE.

According to previous studies of a PET reaction,[12, 33] the pH affects the chemical potential of $OH^-$ and $H_3O^+$ through their configurational entropies, while the absolute potential (i.e. $U_{SHE}$) affects the chemical potential of the electron, $e^-$. As the TS is assumed to have no configurational entropic contributions, its energy only depends on potential via the fractional and pH-independent transfer coefficient $\alpha$. In the case of $H_3O^+$ as the hydrogen source,

$$X^* + H_3O^+ + e^- \rightarrow XH^* + H_2O \quad (5)$$

the activation energy and the reaction energy can be expressed as

$$\begin{aligned}
\Delta G_{a}^{H_3O^+} &= \mu_{TS} + \mu_{\alpha e^-,\,U} - \left(\mu_{H_3O^+} + \mu_{e^-,\,U} + \mu_{X*}\right) \\
&= \mu_{TS} + (\mu_{\alpha e^-,\,0} - \alpha e U_{SHE}) - \left((\mu^0_{H_3O^+} - 2.3k_BT\mathrm{pH}) + \left(\mu_{e^-,\,0} - eU_{SHE}\right) + \mu_{X*}\right) \\
&= \Delta G_{a,\,0}^{H_3O^+} + \beta e U_{SHE} + 2.3k_BT\mathrm{pH} \quad (6) \\
&= \Delta G_{a,\,0}^{H_3O^+} + \beta e U_{RHE} + (1-\beta)2.3k_BT\mathrm{pH} \quad (7)
\end{aligned}$$

$$\begin{aligned}
\Delta G^{H_3O^+} &= \mu_{XH*} + \mu_{H_2O} - \left(\mu_{H_3O^+} + \mu_{e^-,\,U} + \mu_{X*}\right) \\
&= \mu_{XH*} + \mu_{H_2O} + \left((\mu^0_{H_3O^+} - 2.3k_BT\mathrm{pH}) + \left(\mu_{e^-,\,0} - eU_{SHE}\right) + \mu_{X*}\right) \\
&= \Delta G_{0}^{H_3O^+} + eU_{SHE} + 2.3k_BT\mathrm{pH} \quad (8) \\
&= \Delta G_{0}^{H_3O^+} + eU_{RHE} \quad (9)
\end{aligned}$$

In the case of water as the hydrogen source,

$$X^* + H_2O + e^- \rightarrow XH^* + OH^- \quad (10)$$

the activation energy and the reaction energy can be expressed as

$$\Delta G_{\mathrm{a}}^{\mathrm{H_2O}} = \mu_{\mathrm{TS}} + \mu_{\alpha e^-,\,U} - \left(\mu_{\mathrm{H_2O}} + \mu_{e^-,\,U} + \mu_{\mathrm{X*}}\right)$$
$$= \mu_{\mathrm{TS}} + (\mu_{\alpha e^-,\,0} - \alpha e U_{\mathrm{SHE}}) - \left(\mu_{\mathrm{H_2O}} + \left(\mu_{e^-,\,0} - eU_{\mathrm{SHE}}\right) + \mu_{\mathrm{X*}}\right)$$
$$= \Delta G_{\mathrm{a},\,0}^{\mathrm{H_2O}} + \beta e U_{\mathrm{SHE}} \qquad (11)$$
$$= \Delta G_{\mathrm{a},\,0}^{\mathrm{H_2O}} + \beta e U_{\mathrm{RHE}} - \beta 2.3 k_{\mathrm{B}} T \mathrm{pH} \qquad (12)$$
$$\Delta G^{\mathrm{H_2O}} = \mu_{\mathrm{XH*}} + \mu_{\mathrm{OH^-}} - \left(\mu_{\mathrm{H_2O}} + \mu_{e^-,\,U} + \mu_{\mathrm{X*}}\right)$$
$$= \mu_{\mathrm{XH*}} + (\mu_{\mathrm{OH^-}}^{0} - 2.3 k_{\mathrm{B}} T(14 - \mathrm{pH})) - \left(\mu_{\mathrm{H_2O}} + \left(\mu_{\mathrm{e^-},\,0} - eU_{\mathrm{SHE}}\right) + \mu_{\mathrm{X*}}\right)$$
$$= \Delta G_{0}^{\mathrm{H_2O}} + eU_{\mathrm{SHE}} + 2.3 k_{\mathrm{B}} T \mathrm{pH} \qquad (13)$$
$$= \Delta G_{0}^{\mathrm{H_2O}} + eU_{\mathrm{RHE}} \qquad (14)$$

where $\mu_{\mathrm{OH^-}}^{0}$ and $\mu_{\mathrm{H_3O^+}}^{0}$ denote the chemical potentials under the standard conditions, $\mu_{\mathrm{X*}}$ and $\mu_{\mathrm{XH*}}$ the chemical potential of species X* and XH*, $\Delta G_{\mathrm{a},\,0}^{\mathrm{H_2O}}$ / $\Delta G_{0}^{\mathrm{H_2O}}$ the activation energy and reaction energy at 0 V using water as the hydrogen source, $\Delta G_{\mathrm{a}}^{\mathrm{H_3O^+}}$ / $\Delta G_{0}^{\mathrm{H_3O^+}}$ the activation energy and reaction energy at 0 V using $H_3O^+$ as the hydrogen source, $\alpha$ the charge in the TS that gives the potential dependence ($\beta = 1 - \alpha$), $T$ the temperature and $k_{\mathrm{B}}$ the Boltzmann constant.

According to the equations above, the activation energies and reaction energies of acidic and alkaline PET reactions can be schematically illustrated as in **Supplementary Figure 2** at different potential scales. Eq. (6) and Eq. (11) show that the acidic barrier is dependent on both pH and absolute potential while the alkaline barrier only depends on the absolute potential; Eq. (9) and Eq. (14) express that the reaction energies of both acidic and alkaline PET reactions are $U_{\mathrm{RHE}}$-dependent. On the SHE scale, an increased pH leads to an increased acidic barrier by an amount of $2.3k_{\mathrm{B}}T\Delta\mathrm{pH}$, while the alkaline barrier is unaffected by pH. While in RHE scale, since shifts in pH are by definition balanced by shifts in absolute potential (*i.e.* $2.3k_{\mathrm{B}}T\Delta\mathrm{pH} = -e\Delta U$), an increased pH will lead to an increase in acidic barriers by an amount of $(1 - \beta)2.3k_{\mathrm{B}}T\Delta\mathrm{pH}$ and a decrease in barriers from water by an amount of $\beta 2.3k_{\mathrm{B}}T\Delta\mathrm{pH}$.

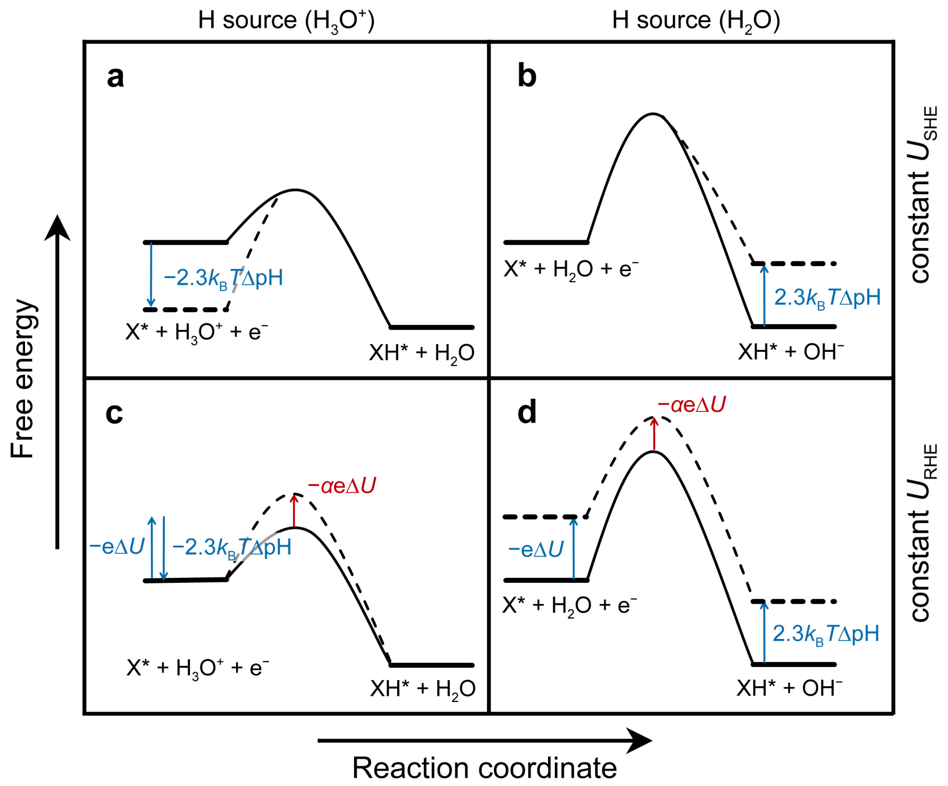


**Supplementary Figure 2. The effects of $H_3O^+$ and $H_2O$ as hydrogen sources on activation/reaction energies of a PET reaction.** Free energy profiles of (**a**) acidic PET with $H_3O^+$ as the hydrogen source and (**b**) alkaline PET with $H_2O$ as the hydrogen source at a constant absolute potential of $U_{SHE}$. Free energy profiles of (**c**) acidic PET with $H_3O^+$ as the hydrogen source and (**d**) alkaline PET with $H_2O$ as the hydrogen source at the same potential of $U_{RHE}$. pH increases from solid profile to dashed profile in each panel. The schematic illustration was conceptualized for the Volmer reaction in Ref.[33].

To correlate the acidic and alkaline barriers using different hydrogen sources, we firstly extrapolated the barriers to a work function of 4.4 eV, which corresponds to 0 V vs. SHE. All TSs were referenced to the IS of aqueous protons and electrons in bulk solution, as determined using the CHE.

According to previous study by Liu *et al.*,[12] there is an energetic difference between the acidic barrier at 0 V vs. RHE at pH = 0 ($\Delta G^{H_3O^+}_{a,\,0\,V_{RHE},\,pH0}$, also equivalent to $\Delta G^{H_3O^+}_{a,\,0\,V_{SHE},\,pH0}$) and the alkaline barrier at 0 V vs. RHE at pH = 14 ($\Delta G^{H_2O}_{a,\,0\,V_{RHE},\,pH14}$) for the Volmer and Heyrovsky reactions (Supplementary Table 3 in Ref.[12]). We therefore refer such an energetic difference as "alkaline barrier correction" as determined by

$$\Delta\Delta G_{alkaline\ corr} = \Delta G^{H_2O}_{a,\,0\,V_{RHE},\,pH14} - \Delta G^{H_3O^+}_{a,\,0\,V_{RHE},\,pH0} \quad (15)$$

According to Eq. (6), Eq. (10), and Eq. (15), we could then correlate the alkaline barrier at 0 V vs. RHE at pH = 7 ($\Delta G^{H_2O}_{a,\,0\,V_{RHE},\,pH7}$) to the acidic barrier at 0 V vs. SHE at pH = 7 ($\Delta G^{H_3O^+}_{a,\,0\,V_{SHE},\,pH7}$) through:

$$\Delta G^{H_2O}_{a,\,0\,V_{RHE},\,pH7} = \Delta G^{H_2O}_{a,\,0\,V_{RHE},\,pH14} - \beta 2.3 k_B T(7-14)$$

$$= (\Delta G^{H_3O^+}_{a,\,0\,V_{RHE},\,pH0} + \Delta\Delta G_{alkaline\ corr}) - \beta 2.3 k_B T(7-14)$$
$$= ((\Delta G^{H_3O^+}_{a,\,0\,V_{SHE},\,pH7} - 2.3 k_B T(7-0)) + \Delta\Delta G_{alkaline\ corr}) - \beta 2.3 k_B T(7-14)$$
$$= \Delta G^{H_3O^+}_{a,\,0\,V_{SHE},\,pH7} + \Delta\Delta G_{alkaline\ corr} - (1-\beta) 2.3 k_B T \times 7 \quad (16)$$

The charge transfer coefficient $\beta$ is determined explicitly from the Bader charge analysis. However, determining the exact $\Delta\Delta G_{alkaline\ corr}$ for each single PET step will be tedious and computational challenging as we discussed above (see Page 8). Therefore, we assume that the effect of altering hydrogen source from $H_3O^+$ to water on other PET reactions is the same as on the Volmer reaction. It has been experimentally measured in Markovic's group that the exchange current density of HER is two to three orders of magnitude lower when operating under alkaline conditions (0.1 M NaOH, pH = 13) compared to acidic conditions (0.1 M $HClO_4$, pH = 1)[31]. Thus, the experimentally determined activation energies are $\Delta H^{H_3O^+}_{a,\,0\,V_{RHE},\,pH1} = 0.19$ eV and $\Delta H^{H_2O}_{a,\,0\,V_{RHE},\,pH13} = 0.48$ eV, respectively. As the TS is assumed to have no entropic contributions, the $\Delta G^{H_3O^+}_{a,\,0\,V_{RHE},\,pH0}$ and $\Delta G^{H_2O}_{a,\,0\,V_{RHE},\,pH14}$ could be determined as 0.21 eV and 0.44 eV based on Eq. (7) and Eq. (12), respectively, if a $\beta$ of 0.65 (the same as in **Supplementary Table 8**) is employed. Therefore, the $\Delta\Delta G_{alkaline\ corr}$ is determined as 0.23 eV using experimentally available data for HER. We then applied such a correction to obtain $\Delta G^{H_2O}_{a,\,0\,V_{RHE},\,pH7}$ according to Eq. (16).

**Supplementary Table 8** lists the estimated $\Delta G^{H_2O}_{a,\,0\,V_{RHE}}$ for protonation steps of $C_2$ species more reduced than CCO*, using an alkaline correction of $\Delta\Delta G_{alkaline\ corr} = 0.23$ eV. All other protonation barriers are the same as reported in our previous work.[5] In Ref.[5], some reported barriers for the formation of $C_1$ species (e.g. CO-H protonation to form COH*) have been compared with barriers reported in previous works using different methods.[34, 35] These barriers were found to align well; therefore, in the present work regarding $C_2$ Oxy/HC formation, we only considered the charge-extrapolation method, which is aiming to be consistent with previous works.[5, 12, 28]

**Supplementary Table 8. Forward activation energies $\Delta G_a$ at $U_{RHE}$ = 0 V for protonation reaction of $C_2$ species more reduced than CCO* on Cu(100).** The charge-transfer coefficient $\beta$ for each protonation step is also provided. $\beta$ was determined by calculating the Bader charge of TS.

| Reaction index | Reaction pathway | Reaction | $\beta$ | $\Delta G_a$ (eV) | |
|---|---|---|---|---|---|
| | | | | pH7 | pH13 |
| 1 | Common | $CCO^* + H^+ + e^- \leftrightarrow CHCO^*$ | 0.50 | 0.78 | 0.60 |
| 2 | Common | $CCO^* + H^+ + e^- \leftrightarrow CCHO^*$ | 0.55 | 1.77 | 1.58 |
| 3 | Common | $CCO^* + H^+ + e^- \leftrightarrow CCOH^*$ | *TS lower than FS* | | |
| 4 | $CH_2CO$ | $CHCO^* + H^+ + e^- \leftrightarrow CH_2CO^{(*)}$ | 0.50 | 0.86 | 0.68 |
| 5 | OCHCH | $CHCO^* + H^+ + e^- \leftrightarrow OCHCH^*$ | 0.45 | 0.97 | 0.81 |
| 6 | CHCOH | $CHCO^* + H^+ + e^- \leftrightarrow CHCOH^*$ | 0.35 | 0.65 | 0.53 |
| 7 | $CH_2CO$ | $CH_2CO^* + H^+ + e^- \leftrightarrow CH_3CO^*$ | 0.45 | 0.41 | 0.25 |
| 8 | $CH_2CO$ | $CH_2CO^* + H^+ + e^- \leftrightarrow OCHCH_2^*$ | 0.50 | 0.47 | 0.29 |

| | | | | | |
|---|---|---|---|---|---|
| 9 | $CH_2CO$ | $CH_2CO^* + H^+ + e^- \leftrightarrow CH_2COH^*$ | *TS lower than FS* | | |
| 10 | OCHCH | $OCHCH^* + H^+ + e^- \leftrightarrow OCHCH_2^*$ | 0.50 | 0.52 | 0.34 |
| 11 | OCHCH | $OCHCH^* + H^+ + e^- \leftrightarrow OCH_2CH^*$ | *unconverged; proceeds through* $OCHCH_2^*$ | | |
| 12 | OCHCH | $OCHCH^* + H^+ + e^- \leftrightarrow CHCHOH^*$ | *TS lower than FS* | | |
| 13 | CHCOH | $CHCOH^* + H^+ + e^- \leftrightarrow CH_2COH^*$ | 0.55 | 0.91 | 0.72 |
| 14 | CHCOH | $CHCOH^* + H^+ + e^- \leftrightarrow CHCHOH^*$ | 0.50 | 0.78 | 0.60 |
| 15 | CHCOH | $CHCOH^* + H^+ + e^- \leftrightarrow CCH^* + H_2O$ | 0.45 | 0.40 | 0.24 |
| 16 | $CH_2CO$ | $CH_3CO^* + H^+ + e^- \leftrightarrow CH_3CHO(l)$ | 0.50 | 0.56 | 0.38 |
| 17 | $CH_2CO$ | $CH_3CO^* + H^+ + e^- \leftrightarrow CH_3COH^*$ | *TS lower than FS* | | |
| 18 | $CH_2CO$ | $CH_3COH^* + H^+ + e^- \leftrightarrow CCH_3^* + H_2O$ | 0.45 | 1.14 | 0.98 |
| 19 | $CH_2CO$ | $CH_2COH^* + H^+ + e^- \leftrightarrow CH_2CHOH(l)$ | 0.50 | 0.87 | 0.69 |
| 20 | $CH_2CO$ | $CH_2COH^* + H^+ + e^- \leftrightarrow CCH_2^* + H_2O$ | 0.45 | 1.21 | 1.05 |
| 21 | OCHCH | $OCHCH_2^* + H^+ + e^- \leftrightarrow CH_2CHOH(l)$ | 0.35 | 0.30 | 0.18 |
| 22 | OCHCH | $OCHCH_2^* + H^+ + e^- \leftrightarrow CH_3CHO(l)$ | 0.40 | 0.37 | 0.23 |
| 23 | OCHCH | $CHCHOH^* + H^+ + e^- \leftrightarrow CH_2CHOH(l)$ | 0.50 | 0.64 | 0.46 |
| 24 | OCHCH | $CHCHOH^* + H^+ + e^- \leftrightarrow CHCH^* + H_2O$ | 0.45 | 1.14 | 0.98 |
| 25 | CHCOH | $CCH^* + H^+ + e^- \leftrightarrow CCH_2^*$ | 0.50 | 0.50 | 0.32 |
| 26 | CHCOH | $CCH^* + H^+ + e^- \leftrightarrow CHCH^*$ | 0.50 | 0.54 | 0.36 |
| 27 | CHCOH | $CCH_2^* + H^+ + e^- \leftrightarrow CHCH_2^*$ | 0.50 | 0.60 | 0.42 |
| 28 | CHCOH | $CCH_2^* + H^+ + e^- \leftrightarrow CCH_3^*$ | 0.50 | 0.77 | 0.59 |
| 29 | CHCOH | $CHCH^* + H^+ + e^- \leftrightarrow CHCH_2^*$ | 0.55 | 0.81 | 0.62 |
| 30 | CHCOH | $CCH_3^* + H^+ + e^- \leftrightarrow CHCH_3^*$ | *unconverged; proceeds through* $H^* + CCH_3^*$ | | |

* one surface site

Despite the above approximation of alkaline barriers, we point out that:

- Despite the higher barriers at the same $U_{RHE}$, water is more likely to be the hydrogen source for protonation reactions at neutral and alkaline conditions due to the abundance of water and the extremely low availability of $H_3O^+$ at these conditions.
- Any underestimation of the alkaline protonation barriers will result in overestimated rate of the reduction step vs. the C–C coupling step starting from the same surface species. This insight should be emphasized especially when we analyze the $C_3$ formation from any of the above $C_2$ intermediates.
- The absolute value of the above barrier is less meaningful than the relative difference between barriers for protonation steps starting from the same surface species (*e.g.* OCCH-H, CHC-HO, and CHCO-H). The relative difference is the major descriptor for the $C_2$ Oxy/HC selectivity.

## Supplementary Note 6: Details of the microkinetic models

Mean-field microkinetic models are simulated with the CATMAP software package.[36] The CatMAP software package used in this work can be accessed and downloaded through https://github.com/SUNCAT-Center/catmap. In our microkinetic models, $CH_4(g)$

was taken as an example of $C_1$ products, $C_2H_4(g)$, $C_2H_2(g)$, $C_2H_6(g)$, $CH_3CHO(g)$, $CH_3CH_2OH(g)$, and $CH_3COOH(g)$ was taken as an example of $C_2$ products, and $H_2(g)$ was included as the main side product. Because of the lack of transport limitation in the model, $OCHCH_2$* simply results in half $CH_3CH_2OH(g)$ and half $CH_3CHO(g)$ in our model at reducing conditions where both the reduction and desorption of $OCHCH_3$* are exothermic. Without the transport limitation of $CH_3CHO(g)$, the desorbed acetaldehyde does not participate in the further reduction to ethanol according to CATMAP's algorithm. To prevent misunderstanding of the acetaldehyde reduction reactions, the formation rate of $CH_3CHO(g)$ was integrated into the formation rate of $CH_3CH_2OH(g)$. Our model is not attempting to address the acetaldehyde/ethanol selectivity. In our previous work,[5] we showed that electrochemical protonation is generally more dominant than surface-mediated hydrogenation. Therefore, we excluded surface hydrogenation steps in our model for simplicity. From the same reason, we did not consider the CHO pathway for $C_1$ and OC-CHO pathway for $C_2$ as we have discussed these to pathways in the prior work.[5] All the elementary steps are described as follows:

$H^+ + e^- + * \leftrightarrow$ H-ele* $\leftrightarrow$ H*
$H^+ + e^- +$ H* $\leftrightarrow$ $H_2$-ele* $\leftrightarrow$ $H_2(g)$ + *
H* + H* $\leftrightarrow$ H-H* + * $\leftrightarrow$ $H_2(g)$ + 2*
OH* + $H^+ + e^- \leftrightarrow$ $H_2O(g)$ + *
CO(g) $\leftrightarrow$ $CO_{HP}$
$CO_{HP}$ + * $\leftrightarrow$ CO*
CO* + $H^+ + e^- \leftrightarrow$ CO-H-ele* $\leftrightarrow$ COH*
COH* + $H^+ + e^- \leftrightarrow$ COH-H-ele* $\leftrightarrow$ C* + $H_2O(g)$
C* + $H^+ + e^- \leftrightarrow$ C-H-ele* $\leftrightarrow$ CH*
CH* + $H^+ + e^- \leftrightarrow$ CH-H-ele* $\leftrightarrow$ $CH_2$*
$CH_2$* + $H^+ + e^- \leftrightarrow$ $CH_2$-H-ele* $\leftrightarrow$ $CH_3$*
$CH_3$* + $H^+ + e^- \leftrightarrow$ $CH_3$-H-ele* $\leftrightarrow$ $CH_4(g)$ + *
2CO* $\leftrightarrow$ OCCO**
OCCO** + $H^+ + e^- \leftrightarrow$ OCCO-H-ele** $\leftrightarrow$ OCCOH**
OCCOH** + $H^+ + e^- \leftrightarrow$ CCO* + $H_2O(g)$ + *
CO* + C* $\leftrightarrow$ OC-C** $\leftrightarrow$ CCO* + *
$CO_{HP}$ + C* $\leftrightarrow$ C-CO* $\leftrightarrow$ CCO*
CCO* + $H^+ + e^- \leftrightarrow$ OCC-H-ele* $\leftrightarrow$ CHCO*
[The above reaction mechanism is directly adopted from Ref.[5].]

CHCO* + $H^+ + e^- \leftrightarrow$ OCCH-H-ele* $\leftrightarrow$ $CH_2CO(g)$ + *
$CH_2CO$** $\leftrightarrow$ $CH_2CO(g)$ + 2*
$CH_2CO(g)$ $\leftrightarrow$ $CH_2CO$-$H_2O$ $\leftrightarrow$ $CH_3COOH(g)$
$CH_2CO$** + $H^+ + e^- \leftrightarrow$ H-$CH_2CO$-ele** $\leftrightarrow$ $CH_3CO$**
$CH_3CO$** + $H^+ + e^- \leftrightarrow$ $CH_3C$-HO-ele** $\leftrightarrow$ $OCHCH_3$* + *
$CH_2CO$** + $H^+ + e^- \leftrightarrow$ $CH_2C$-HO-ele** $\leftrightarrow$ $OCHCH_2$**

[The above reaction mechanism represents the major $CH_2CO$ pathway; $CH_2CO$-$H_2O$ refers to a TS constructed using an experimental ethenone hydration barrier measured by Bothe *et al.*[37].]

CHCO* + $H^+ + e^- +$ * $\leftrightarrow$ CHC-HO-ele* + * $\leftrightarrow$ OCHCH**

$OCHCH^{**} + H^+ + e^- \leftrightarrow OCHCH\text{-}H\text{-}ele^{**} \leftrightarrow OCHCH_2^{**}$
$OCHCH_2^{**} + H^+ + e^- \leftrightarrow OCHCH_2\text{-}H\text{-}ele^* + * \leftrightarrow OCHCH_3^* + *$
$OCHCH_3^* \leftrightarrow CH_3CHO(g) + *$
$OCHCH_2^{**} + H^+ + e^- \leftrightarrow CH_2CHO\text{-}H\text{-}ele^{**} \leftrightarrow CH_2CHOH^{**}$
$CH_2CHOH^{**} \leftrightarrow CH_2CHOH(g) + 2*$
$CH_2CHOH(g) \leftrightarrow CH_2C\text{-}HOH \leftrightarrow CH_3CHO(g)$
$OCHCH_3^* + H^+ + e^- \leftrightarrow OCH_2CH_3^*$
$OCH_2CH_3^* + H^+ + e^- \leftrightarrow CH_3CH_2OH(g) + *$

[The above reaction mechanism represents the major OCHCH pathway; $CH_2C$-HOH refers to a TS constructed using a diabetic tautomerization barrier assessed by Clark *et al.*[38].]

$CHCO^* + H^+ + e^- + * \leftrightarrow CHCO\text{-}H\text{-}ele^* + * \leftrightarrow CHCOH^{**}$
$CHCOH^{**} + H^+ + e^- \leftrightarrow CHCOH\text{-}H\text{-}ele^{**} \leftrightarrow CCH^{**} + H_2O(g)$
$CCH^{**} + H^+ + e^- \leftrightarrow CCH\text{-}H\text{-}ele^* + * \leftrightarrow CCH_2^* + *$
$CCH_2^* + H^+ + e^- \leftrightarrow H\text{-}CCH_2\text{-}ele^* \leftrightarrow CHCH_2^*$
$CHCH_2^* + H^+ + e^- \leftrightarrow C_2H_4(g) + *$

[The above reaction mechanism represents the major CHCOH pathway leading to ethylene.]

$CCH^{**} + H^+ + e^- \leftrightarrow H\text{-}CCH\text{-}ele^{**} \leftrightarrow CHCH^{**}$
$CHCH^{**} \leftrightarrow C_2H_2(g) + 2*$
$CHCH^{**} + H^+ + e^- \leftrightarrow H\text{-}CHCH\text{-}ele^{**} \leftrightarrow CHCH_2^* + *$
$CCH_2^* + H^+ + e^- \leftrightarrow CCH_2\text{-}H\text{-}ele^* \leftrightarrow CCH_3^*$
$CCH_3^* + H^+ + e^- \leftrightarrow CHCH_3^*$
$CHCH_3^* + H^+ + e^- \leftrightarrow CH_2CH_3^*$
$CH_2CH_3^* + H^+ + e^- \leftrightarrow C_2H_6(g) + *$

[The above reaction mechanism represents the minor CHCOH pathway leading to acetylene and ethane.]

(* one surface site; ** two surface sites; HP: Helmholtz plane)

Note that all above reactions actually use alkaline barriers of $\Delta G^{H_2O}_{a,\,0\,V_{RHE}}$ shown in **Supplementary Table 8** and the alkaline protonation step of $X^* + H_2O + e^- \rightarrow XH^* + OH^-$ is written as the corresponding acidic form of $X^* + H^+ + e^- \rightarrow XH^*$ to fit with the CATMAP software package, in which the CHE model is implemented in to correct the energies of proton and electron at a RHE scale. And the actual concentration of $H^+$ is defined to be the same as water probability [$H_2O$] of 1.

Adsorbate-adsorbate interactions are considered for all possible reaction intermediate pairs. The following equations are adopted to describe the adsorption energy as a function of coverage[39, 40]

$$E_i(\theta_i) = \begin{cases} E_i^0 & \text{when } |\theta| \leqslant \theta_0 \\ E_i^0 + \sum_j f\varepsilon_{ij}\theta_j & \text{when } |\theta| > \theta_0 \end{cases} \quad (17)$$

where $E_i(\theta_i)$ denotes the differential adsorption energy of species i at coverage $\theta_i$, $E_i^0$ the differential adsorption energy at low coverage limit, $\theta_0$ the threshold coverage (0.25 monolayer (ML) in this work), $|\theta|$ the sum of the surface coverages of all adsorbates except H* (the H* coverage is excluded to account for H* being much smaller than CO and therefore has little effect on determining the strength of the interactions), $\varepsilon_{ij}$ the cross-interaction parameter between species i and j, $f$ the fractional coverage, which can be calculated as $f = \frac{|\theta| - \theta_0}{|\theta|}$.

As shown above, the interactions are significant only when adsorbate coverages exceed a threshold of about 0.25 ML, and CO* is the only intermediate that has a coverage above this threshold in the potential range of interest, therefore only the interactions between CO* and other intermediates affect the energetics. The energetics of intermediates and TSs are therefore all functions of coverage.

The barriers and reaction energies could thus shift accordingly at high surface coverage. We have assumed the interaction parameters to be the same due to the similar sizes of molecules. Recently, the CO coverage effect has been demonstrated on tuning the $C_2$ Oxy/HC selectivity.[41] Theoretical consideration of such a coverage effect on key $C_2$ intermediates such as CHCOH*, OCHCH*, and $CH_2CO$* should be a future direction.

The adsorbate cross-interaction parameters were listed below.

$\epsilon_{CO*, CO*} = 2.47$
$\epsilon_{CO*, H*} = 0.73$
$\epsilon_{CO*, H\text{-}ele*} = 0.79$
$\epsilon_{CO*, H2\text{-}ele*} = 0.51$
$\epsilon_{CO*, H\text{-}H*} = 1.16$
$\epsilon_{CO*, OH*} = 1.60$
$\epsilon_{OH*, OH*} = 1.03$

All other species were assumed to have same interactions as those of CO*. All unlisted $\epsilon_{i,j}$ are assumed to be zero.

**Supplementary Note 7: Details of the degree of selectivity control (DSC) analysis**

The DSC is a concept derived from the degree of rate control (DRC), which was conceptualized by Campbell *et al.*[42, 43] The DRC is a mathematical approach for analyzing reaction mechanisms and kinetics of multistep reactions. The DRC for elementary step $i$, $X_{RC,i}$, is initially defined as

$$X_{RC,i} = \frac{k_i}{r}\left(\frac{\partial r}{\partial k_i}\right)_{k_{j\neq i}, K_i} = \left(\frac{\partial \ln r}{\partial \ln k_i}\right)_{k_{j\neq i}, K_i} \quad (18)$$

where $r$ is the net reaction rate to the product of interest, and the partial derivative is taken holding constant the rate constants, $k_j$, for all other steps $j \neq i$ and the equilibrium constant, $K_i$, for step $i$ (and all other steps too, since their forward and reverse rate constants are held fixed). By this definition above, $X_{RC,i}$ equals the relative increase in the net rate per relative increase in the rate constant for step $i$ (differentially). The larger the numeric value of $X_{RC,i}$ is for a given step, the bigger is the influence of its rate constant on

the overall reaction rate $r$. A positive value indicates that increasing $k_i$ will increase the net rate $r$; such steps are termed rate-limiting steps. A negative value indicates the opposite; such steps are termed inhibition steps. While Eq. (18) looks like equations used in standard differential sensitivity analyses, it ensures thermodynamic and kinetic consistency as the entities in this partial derivative are held constant. By further correlating $k_i$ to reaction thermodynamics and kinetics using transition state theory, $X_{\mathrm{RC},i}$ could be expressed in a more general way, the generalized degree of rate control of species $i$, $\mathrm{DRC}_i$, as:

$$\mathrm{DRC}_i = \frac{1}{r}\left(\frac{\partial r}{\partial\left(\frac{-G_i^0}{RT}\right)}\right)_{G_{j\neq i}^0} = \left(\frac{\partial \ln r}{\partial\left(\frac{-G_i^0}{RT}\right)}\right)_{G_{j\neq i}^0} = \left(\frac{-\partial \ln r}{\partial\left(\frac{G_i^0}{RT}\right)}\right)_{G_{j\neq i}^0} \quad (19)$$

where the partial derivative is now taken holding constant the standard-state free energy of all other species (intermediates, transition states, reactants, and products), $j$. Its value describes the relative increase in net rate due to the (differential) stabilization of the standard-state free energy for species $i$, holding all the other species' energies constant. Thus, Eq. (19) probes the importance of one species' free energy in the full standard-state free-energy surface for the full reaction.

Similarly, the generalized DSC of species $i$, $\mathrm{DSC}_i$, could be defined as the sensitivity of the selectivity, $S = r_\mathrm{P}/r_\mathrm{R}$, where $r_\mathrm{P}$ is the rate of production of the desired product P and $r_\mathrm{R}$ is the rate of consumption of a reactant R, to energies of $i$ as:

$$\mathrm{DSC}_i = \frac{1}{S}\left(\frac{\partial S}{\partial\left(\frac{-G_i^0}{RT}\right)}\right)_{G_{j\neq i}^0} = \left(\frac{\partial \ln S}{\partial\left(\frac{-G_i^0}{RT}\right)}\right)_{G_{j\neq i}^0} = \left(\frac{-\partial \ln S}{\partial\left(\frac{G_i^0}{RT}\right)}\right)_{G_{j\neq i}^0}$$

$$= \left(\frac{-\partial \ln(r_\mathrm{P}/r_\mathrm{R})}{\partial\left(\frac{G_i^0}{RT}\right)}\right)_{G_{j\neq i}^0} = \left(\frac{-\partial \ln(r_\mathrm{P})}{\partial\left(\frac{G_i^0}{RT}\right)}\right)_{G_{j\neq i}^0} - \left(\frac{-\partial \ln(r_\mathrm{R})}{\partial\left(\frac{G_i^0}{RT}\right)}\right)_{G_{j\neq i}^0}$$

$$= \mathrm{DRC}_{i,\mathrm{P}} - \mathrm{DRC}_{i,\mathrm{R}} \quad (20)$$

where $\mathrm{DRC}_{i,\mathrm{P}}$ and $\mathrm{DRC}_{i,\mathrm{R}}$ are the degrees of rate control of species $i$ for the rates of making P and consuming R, respectively. The value of $\mathrm{DSC}_i$ describes the relative increase in net selectivity to P from R due to the (differential) stabilization of the standard-state free energy for species $i$ holding all other species' energies constant. The DSCs shown in **Figure 4** are all calculated using ($\mathrm{DRC}_{i,\mathrm{P}}$ - $\mathrm{DRC}_{i,\mathrm{CO}}$) where P is ethylene, ethanol, or acetic acid. The $\mathrm{DRC}_{i,\mathrm{P}}$ and $\mathrm{DRC}_{i,\mathrm{CO}}$ were mathematically obtained using the code implemented in the CATMAP software package.[36]

## Supplementary Note 8: Derivation of the analytical expression of relative rates of $C_2$ Oxy vs. $C_2$ HC

As shown in **Figure 4a–c**, the selectivity-determining step (SDS) changes with pH and potentials. We then derived a simple analytic expression of the $C_2$ Oxy/HC molecular ratio as

$$\frac{r_{C_2\ \mathrm{Oxy}}}{r_{C_2\ \mathrm{HC}}} = \frac{A\theta_{\mathrm{CHCO*}}[\mathrm{H_2O}]\exp\left(-\frac{\Delta G_\mathrm{a}^{\mathrm{OCCH\text{-}H}}}{k_\mathrm{B}T}\right) + A\theta_{\mathrm{CHCO*}}[\mathrm{H_2O}]\exp\left(-\frac{\Delta G_\mathrm{a}^{\mathrm{CHC\text{-}HO}}}{k_\mathrm{B}T}\right)}{A\theta_{\mathrm{CHCO*}}[\mathrm{H_2O}]\exp\left(-\frac{\mathrm{Max}(\Delta G_\mathrm{a}^{\mathrm{CHCO\text{-}H}}, \Delta G_\mathrm{rxn}^{\mathrm{CHCOH}}, \Delta G_\mathrm{a}^{\mathrm{CHCOH\text{-}H}})}{k_\mathrm{B}T}\right)} \tag{1}$$

where $r_{C_2\ \mathrm{Oxy}}$ and $r_{C_2\ \mathrm{HC}}$ are the formation rate of $C_2$ Oxy and HC, respectively, $A$ the pre-exponential term that is assumed to be the same, $\theta_{\mathrm{CHCO*}}$ the coverage of CHCO*, [$H_2O$] the probability of water as the proton source at the interface, $\Delta G_\mathrm{a}$ and $\Delta G_\mathrm{rxn}$ the activation energy and reaction energy relative to CHCO*, $k_\mathrm{B}$ the Boltzmann constant, and $T$ the temperature. On Cu(100), OCCH-H$^{\mathrm{TS}}$ is lower in energy than CHC-HO$^{\mathrm{TS}}$ by at least 0.11 eV, which further result in around two orders of magnitude difference between absolute values of the first and the second term in numerator of Eq. (1) when $T$ = 300K , and thus the above expression could be further simplified as

$$\ln\left(\frac{r_{C_2\ \mathrm{Oxy}}}{r_{C_2\ \mathrm{HC}}}\right) \approx \frac{-\Delta G_\mathrm{a}^{\mathrm{OCCH\text{-}H}} + \mathrm{Max}(\Delta G_\mathrm{a}^{\mathrm{CHCO\text{-}H}}, \Delta G_\mathrm{rxn}^{\mathrm{CHCOH}}, \Delta G_\mathrm{a}^{\mathrm{CHCOH\text{-}H}})}{k_\mathrm{B}T} \tag{2}$$

At the left leg where $\Delta G_\mathrm{a}^{\mathrm{CHCOH\text{-}H}} > \Delta G_\mathrm{a}^{\mathrm{CHCO\text{-}H}}$,

$$\begin{aligned}\ln\left(\frac{r_{C_2\ \mathrm{Oxy}}}{r_{C_2\ \mathrm{HC}}}\right) &\approx \frac{-\Delta G_\mathrm{a}^{\mathrm{OCCH\text{-}H}} + \Delta G_\mathrm{a}^{\mathrm{CHCOH\text{-}H}}}{k_\mathrm{B}T} \\ &= \frac{-(\Delta G_{\mathrm{a,\,0}}^{\mathrm{OCCH\text{-}H}} - \beta^{\mathrm{OCCH\text{-}H}} eU_{\mathrm{RHE}}) + (\Delta G_{\mathrm{a,\,0}}^{\mathrm{CHCOH\text{-}H}} - (1+\beta^{\mathrm{CHCOH\text{-}H}})eU_{\mathrm{RHE}})}{k_\mathrm{B}T} \\ &= \frac{(\beta^{\mathrm{OCCH\text{-}H}} - \beta^{\mathrm{CHCOH\text{-}H}} - 1)eU_{\mathrm{RHE}} + (\Delta G_{\mathrm{a,\,0}}^{\mathrm{CHCOH\text{-}H}} - \Delta G_{\mathrm{a,\,0}}^{\mathrm{OCCH\text{-}H}})}{k_\mathrm{B}T}\end{aligned} \tag{3}$$

while at the right leg where $\Delta G_\mathrm{a}^{\mathrm{CHCOH\text{-}H}} < \Delta G_\mathrm{a}^{\mathrm{CHCO\text{-}H}}$,

$$\begin{aligned}\ln\left(\frac{r_{C_2\ \mathrm{Oxy}}}{r_{C_2\ \mathrm{HC}}}\right) &\approx \frac{-\Delta G_\mathrm{a}^{\mathrm{OCCH\text{-}H}} + \Delta G_\mathrm{a}^{\mathrm{CHCO\text{-}H}}}{k_\mathrm{B}T} \\ &= \frac{-(\Delta G_{\mathrm{a,\,0}}^{\mathrm{OCCH\text{-}H}} - \beta^{\mathrm{OCCH\text{-}H}} eU_{\mathrm{RHE}}) + (\Delta G_{\mathrm{a,\,0}}^{\mathrm{CHCO\text{-}H}} - \beta^{\mathrm{CHCO\text{-}H}} eU_{\mathrm{RHE}})}{k_\mathrm{B}T} \\ &= \frac{(\beta^{\mathrm{OCCH\text{-}H}} - \beta^{\mathrm{CHCO\text{-}H}})eU_{\mathrm{RHE}} + (\Delta G_{\mathrm{a,\,0}}^{\mathrm{CHCO\text{-}H}} - \Delta G_{\mathrm{a,\,0}}^{\mathrm{OCCH\text{-}H}})}{k_\mathrm{B}T}\end{aligned} \tag{4}$$

The Eq. (3) and (4) provides quantitative assessments of the slope and intercepts of each leg in **Figure 3c**. Note that Eq. (3) and Eq. (4) were obtained based on the TS energies on Cu(100). For other Cu facets or materials featuring a different dominant $C_2$ Oxy pathway (*e.g.* the OCHCH pathway on Cu(110) as indicated in **Figure 5**), $\Delta G_{\mathrm{a,\,0}}^{\mathrm{OCCH\text{-}H}}$ and $\beta^{\mathrm{OCCH\text{-}H}}$ in Eq. (3) and Eq. (4) should be replaced by $\Delta G_{\mathrm{a,\,0}}^{\mathrm{CHC\text{-}HO}}$ and $\beta^{\mathrm{CHC\text{-}HO}}$, respectively.

When we consider the change in local pH induced by either significant consumption of $CO_2$ under high current densities at neutral conditions[44, 45] or the neutralization reaction between $CO_2$ and $OH^-$ under low current densities at alkaline conditions,[46] the TS energies of the first protonation step of CHCO* ($\Delta G_{a,0}^{\text{OCCH-H}}$, $\Delta G_{a,0}^{\text{CHC-HO}}$, and $\Delta G_{a}^{\text{CHCO-H}}$) will not be affected due to the $U_{\text{SHE}}$-dependence of alkaline protonation barriers (discussed in **Supplementary Note 5** and previous works[5, 12]). However, the reaction energy, specifically $\Delta G_{\text{rxn}}^{\text{CHCOH}}$, and the TS energy of the second protonation step. *i.e.* $\Delta G_{a}^{\text{CHCOH-H}}$ will increase by an amount of $2.3k_{\text{B}}\text{T}\Delta\text{pH}$ (0.059ΔpH at 298 K) due to the presence of $OH^-$ of higher concentration at the interface than in the bulk solution (**Supplementary Figure 13**). At the meantime, the absolute potential of the working electrode will not be affected since it is referenced to the potential of reference electrode that does not undergo a local pH change. Therefore, the Eq. (3) could then be re-written as

$$\ln\left(\frac{r_{\text{C}_2\text{ Oxy}}}{r_{\text{C}_2\text{ HC}}}\right) \approx \frac{(\beta^{\text{OCCH-H}} - \beta^{\text{CHCOH-H}} - 1)eU_{\text{RHE}} + (\Delta G_{a,0}^{\text{CHCOH-H}} - \Delta G_{a,0}^{\text{OCCH-H}})}{k_{\text{B}}T} + 2.3\Delta\text{pH} \quad (5)$$

where ΔpH refers to the local pH change.

## Supplementary Note 9: The underlying limitations/assumptions from our methodology

Put succinctly, the underlying limitations/assumptions from our methodology that we cannot fully address are as followed:

- Our TS complexes are found with CI-NEB, which allow water molecules ample time to relax to their ground state even though PET steps are measured to be very fast.
- We assume that the first water layer is arranged in an ice-like manner. The water layer can be obtained via minima-hopping. Under reducing conditions, the water molecules face down with hydrogens pointed towards the surface.
- The CHE model inherently assumes electron transfer is in sync with each other; we do not consider steps like one-electron-two-proton transfer. A notable limitation to this assumption is the inability to model intermediates with a charged state such as a $CO_2^-$.[47, 48]
- We assume Cu is not in a positive oxidation state.
- We assume cations and water molecules do not adsorb on the surface.
- We assume that the TS complex found via protonation using $H_3O^+$ is similar to that with protonation using $H_2O$, such that a constant correction scheme can be used as an extrapolation.
- We assume that an electrochemical interface can be modeled as a plate capacitor. This allows us to separate the energy contribution from chemical bonding and electrostatics.
- We assume that electrification and solvation effects are entirely independent from each other; the calculations of electrification only model the intermediates on a bare slab.
- Under Bader charge partitioning of the TS complex, the charge of the transferring proton is *not* considered part of the TS complex. This is despite the fact that we consider the transferred proton in the TS complex when computing vibrational modes.

We believe that as long as future works subscribe to these assumptions, the findings of this work are upheld.[49] We note that these assumptions have been largely successful attaining microkinetic models that can model the product rates and distributions on Cu.[5, 12, 28] Competing mechanistic models that pick apart these assumptions will also need to show their capabilities to account for product rates and distributions across pH and applied potentials.

In addition, we would like to clarify herein that while accurate theoretical simulation of electrochemical activation barriers is inherently difficult using DFT-based approaches, there are new methods recently developed to capture the general trends in protonation barriers,[49] or to explicitly include thermal fluctuations of the solvent at the electrochemical interface.[50] Revisiting the SDSs we identified in this work using these methods is definitely one of the future works worth being investigated.

# 2. Supplementary Figures and Tables
### (Relevant to the Contents in the Main Text)

## 2.1. Reaction pathways prior to CCO* controlling the total rate

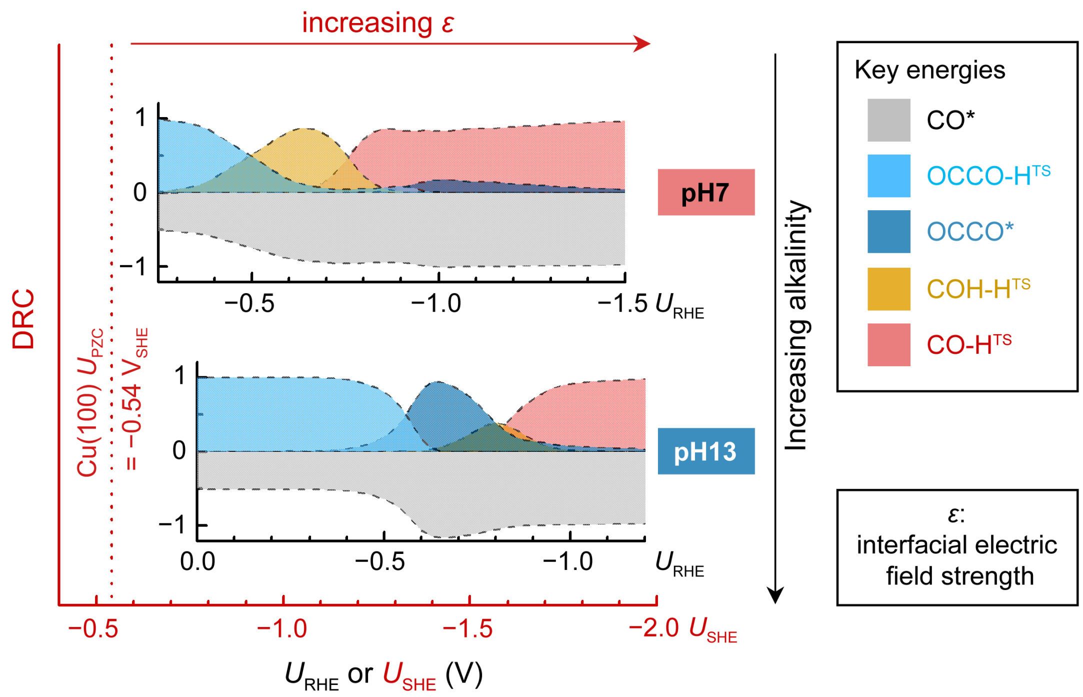


**Supplementary Figure 3. Degree of rate control (DRC) analysis.** Simulated DRC of COR at bulk pH7 and bulk pH13, which was directly adopted from Ref.[5]. As conceptualized by Campbell *et al.*,[42, 43] DRC serves as a powerful tool to quantify the magnitude of rate controlling by a certain intermediate or a TS. A positive (negative) value of DRC indicates that the corresponding reaction intermediate or TS needs to be stabilized (destabilized) in order to enhance the rate. The boundary values of 1 and −1 represent full rate control by the intermediates. Key intermediates and TSs are shown in light gray (CO*), azure (OCCO-H$^{TS}$), blue (OCCO*), yellow (COH-H$^{TS}$), and red (CO-H$^{TS}$), respectively. OCCO-H$^{TS}$, COH-H$^{TS}$, and CO-H$^{TS}$ refer to TSs of OCCO-H, COH-H, and CO-H protonation steps, respectively. This figure clearly shows that the rate-determining step of COR is initially OCCO-H protonation and then changes into COH-H protonation and CO-H protonation as the potential goes negative at pH7. *Videlicet*, the dominant COR pathway shifts from the OCCOH to the COH/OC-C with increasing overpotential. At pH13, the change in RDS follows the same trend but the $U_{SHE}$ for the pathway transition becomes more negative.

## 2.2. Reaction pathways for COR bifurcating from CCO* towards $C_2$ Oxy/HC

### *2.2.1. CCO*/CHCO**

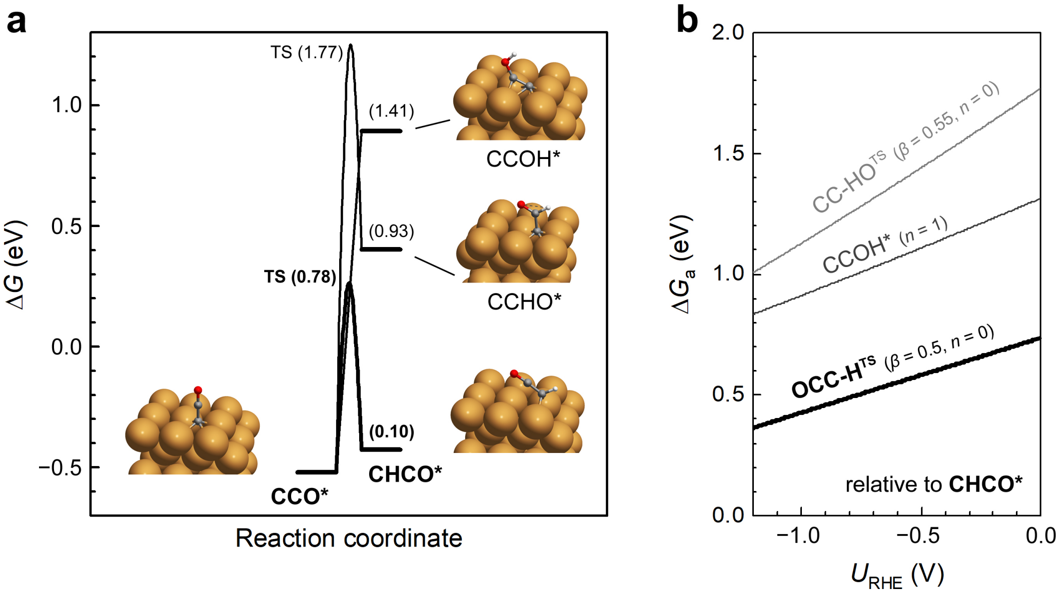


**Supplementary Figure 4. Energetic of CCO protonation on Cu(100) at pH7.** (**a**) FEDs at $U_{RHE}$ = 0 V. The protonation steps of surface-bound α-C or the dangling carbonyl bonds to form CHCO*, CCHO*, and CCOH* were considered. Solvation corrections were applied and all the energies were referenced to CO(g), $H_2$(g), and $H_2O$(g) according to the CHE model. Numbers in the parentheses represent the $\Delta G_{rxn}$ or $\Delta G_a$ relative to CCO*. To simply the diagrams, field corrections were not applied. The optimized geometries of adsorbates on Cu(100) are shown as insets. (**b**) $U$-dependent $\Delta G_a$ of three protonation steps of CCO*. The differences in $\beta$ and $n$ (the number of electron and proton transferred to CCO* before the corresponding TS) and dependency on interfacial field were explicitly considered. $\Delta G_a$ was relative to CCO*. Clearly, the OCC-H protonation toward CHCO* is more dominant than the other two steps in a wide potential range.

According to **Supplementary Figure 4**, the reaction Gibbs free energy ($\Delta G_{rxn}$) and the forward activation barrier ($\Delta G_a$) of H-CCO protonation are ~1.0 eV lower than the $\Delta G_{rxn}$ and $\Delta G_a$ for the CC-HO protonation. Likewise, the CCO-H protonation is mainly limited by the very high $\Delta G_{rxn}$, which is at least 0.5 eV higher than the $\Delta G_a$ of H-CCO protonation at all potentials. The negligible backward barrier ($\Delta G_b$) of CCO-H protonation further verifies the transient nature of CCOH* on the Cu(100) surface.

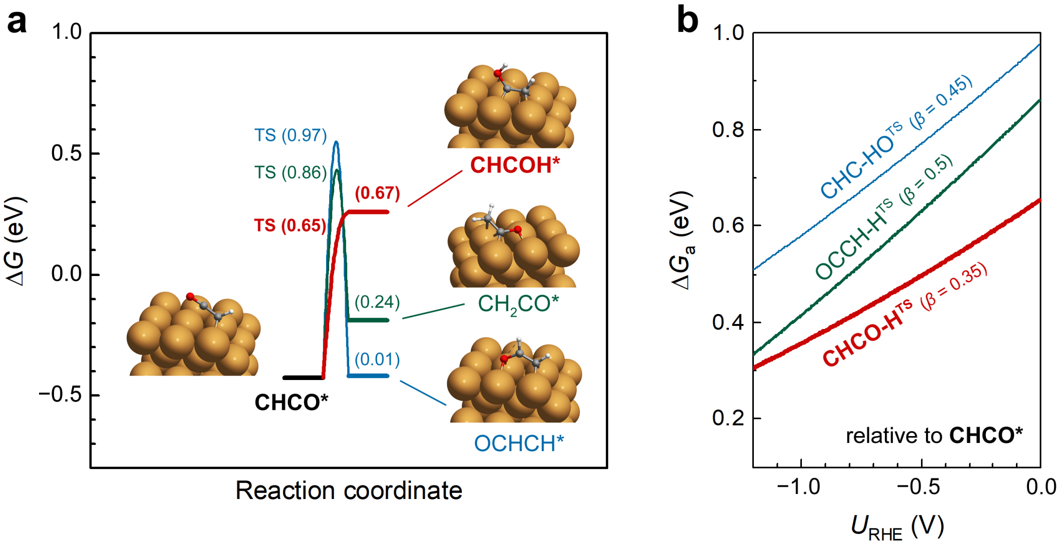


**Supplementary Figure 5. Energetic of CHCO protonation on Cu(100) at pH7.** (**a**) FEDs at $U_{\mathrm{RHE}}$ = 0 V. The protonation steps of surface-bound α-C or the dangling carbonyl bonds to form $CH_2CO$* (green), OCHCH* (blue), and CHCOH* (red) were considered. OCHCH* was found to be stabilized by 0.81 eV with the formation of a surface–O bond when compared to CHCHO* with the carbonyl bond sticking out. Solvation corrections were applied and all the energies were referenced to CO(g), $H_2$(g), and $H_2O$(g) according to the CHE model. Numbers in the parentheses represent the $\Delta G_{\mathrm{rxn}}$ or $\Delta G_{\mathrm{a}}$ relative to CHCO*. To simply the diagrams, field corrections were not applied. The optimized geometries of adsorbates on Cu(100) are shown as insets. Note that at $U_{\mathrm{RHE}}$ = 0 V, the CHCOH formation is limited by the $\Delta G_{\mathrm{rxn}}$ (0.67 eV) rather than $\Delta G_{\mathrm{a}}$ (0.65 eV). Because of the larger magnitude of $\Delta G_{\mathrm{rxn}}$ than $\Delta G_{\mathrm{a}}$ shifting towards negative with increasing overpotential, the CHCO-H step is controlled by the $\Delta G_{\mathrm{a}}$ at negative potentials. (**b**) $U$-dependent $\Delta G_{\mathrm{a}}$ of three protonation steps of CHCO*. The differences in $\beta$ and dependency on interfacial field were explicitly considered. $\Delta G_{\mathrm{a}}$ was relative to CHCO*.

*2.2.2. The CHCOH pathway*

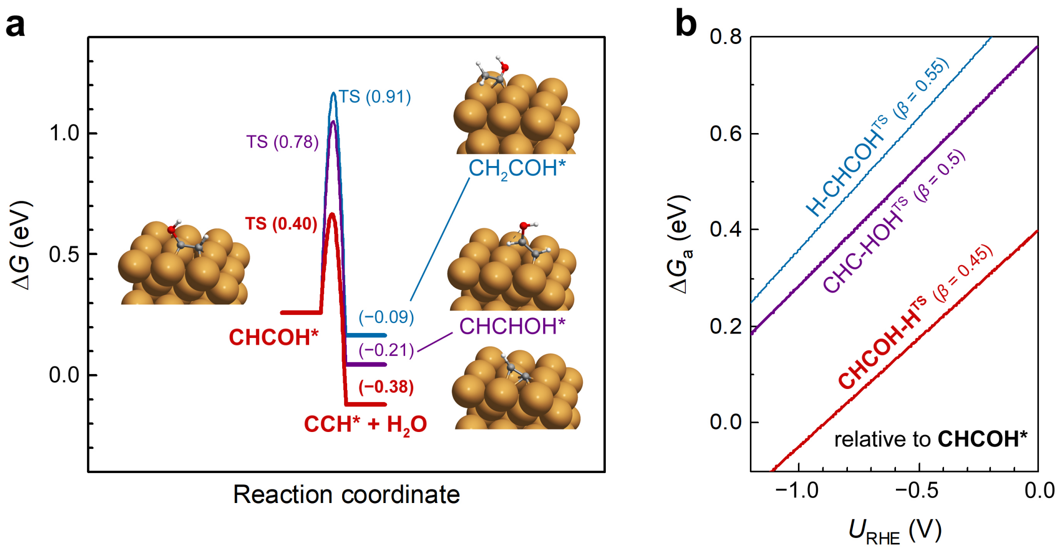


**Supplementary Figure 6. Energetic of CHCOH protonation on Cu(100) at pH7.** (**a**) FEDs at $U_{RHE}$ = 0 V. The protonation steps of α-C, hydroxylated C, or the hydroxyl group to form $CH_2COH^*$ (blue), CHCHOH* (purple), or CCH* + $H_2O$ (red) were considered. Solvation corrections were applied and all the energies were referenced to CO(g), $H_2$(g), and $H_2O$(g) according to the CHE model. Numbers in the parentheses represent the $\Delta G_{rxn}$ or $\Delta G_a$ relative to CHCOH*. To simply the diagrams, field corrections were not applied. The optimized geometries of adsorbates on Cu(100) are shown as insets. (**b**) $U$-dependent $\Delta G_a$ of three protonation steps of CHCOH*. The differences in $\beta$ and dependency on interfacial field were explicitly considered. $\Delta G_a$ was relative to CHCOH*. Despite the smaller $\beta$, the CHCOH-H protonation toward a HC specie is generally more dominant than the other two steps leading Oxy species.

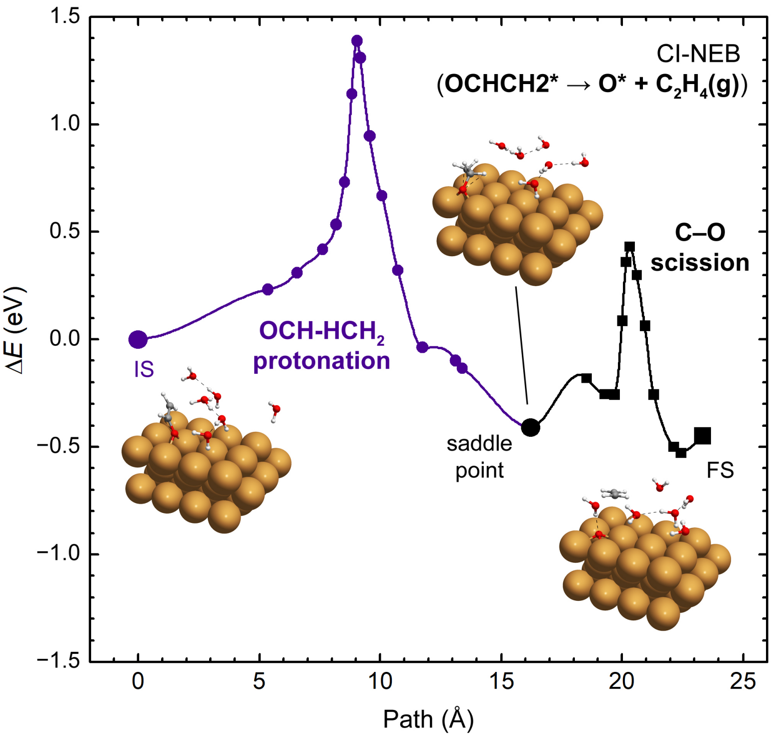


**Supplementary Figure 7. CI-NEB analysis of $OCHCH_2$ reduction step to O* + $C_2H_4$(g) on Cu(100).** The CI-NEB profile shown herein was converged and exhibited two TSs as indicated in **Figure 2g** as TS6 and TS7. The *y*-axis shows the raw electronic energies before charge extrapolation and thus the energy fluctuation cannot reflect the actual barrier under constant-potential conditions. Snapshots of several states marked in the profile were shown as insets. It is clearly shown that the $OCHCH_2$* presents at a saddle point along the CI-NEB profile and therefore such a process is not concerted.

*2.2.3. The OCHCH pathway*

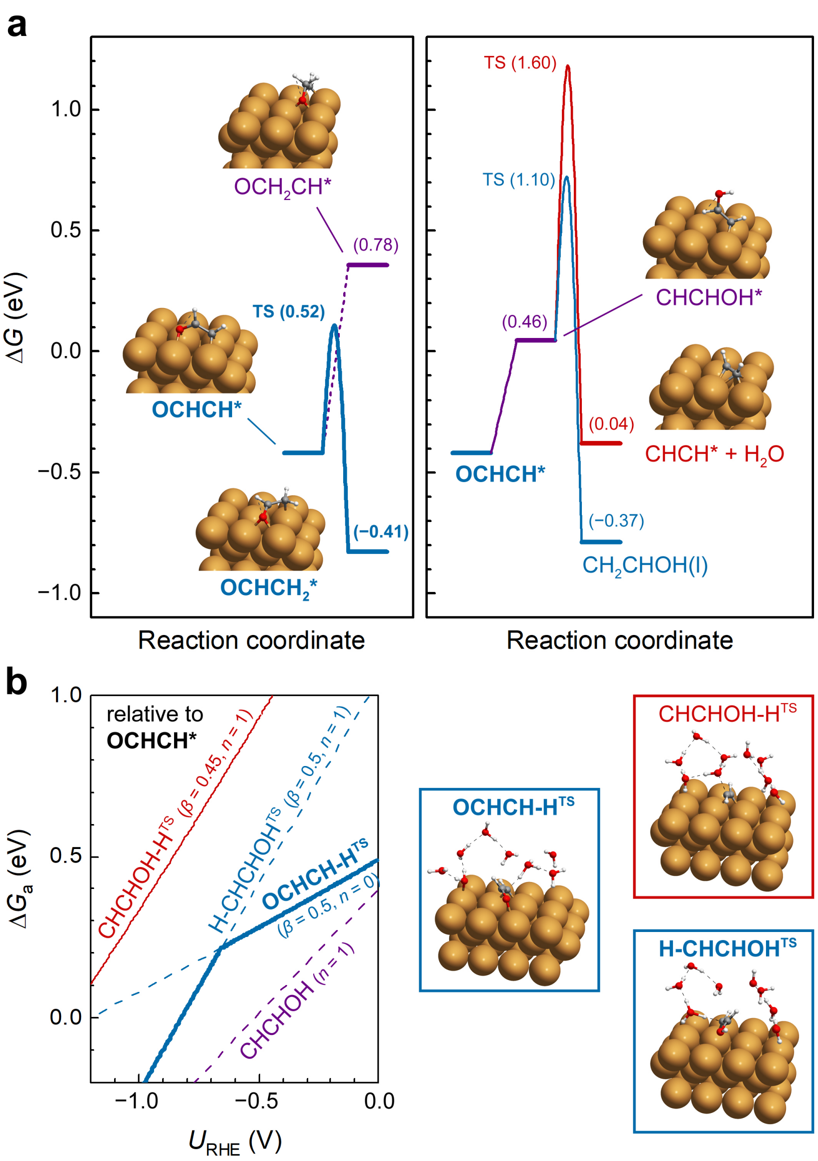


**Supplementary Figure 8. Energetic of OCHCH protonation on Cu(100) at pH7.** (**a**) FEDs at $U_{\mathrm{RHE}}$ = 0 V. The protonation steps of surface-bound C, bridge C, or surface-bound O to form $OCHCH_2$* (blue), $OCH_2CH$* (purple, dashed), and CHCHOH* (purple, solid) were considered. Further reduction steps of CHCHOH* to CHCH* + $H_2O$ (red) or $CH_2CHOH$(l) (blue) were also considered since the CHCHOH formation from OCHCH* is shown to be kinetically facile. Solvation corrections were applied and all the energies were referenced to CO(g), $H_2$(g), and $H_2O$(g) according to the CHE model. Numbers in the parentheses represent the $\Delta G_{\mathrm{rxn}}$ or $\Delta G_{\mathrm{a}}$ relative to OCHCH*. To simply the diagrams, field corrections were not applied. The optimized geometries of adsorbates on Cu(100) are shown as insets. (**b**) $U$-dependent $\Delta G_{\mathrm{a}}$ of several protonation steps for OCHCH* and CHCHOH*. The differences in $\beta$ and $n$ (the number of electron and proton transferred to OCHCH* before the corresponding TS) and dependency on interfacial field were explicitly considered. $\Delta G_{\mathrm{a}}$ was relative to OCHCH*. TS structures of OCHCH-H, CHCHOH-H, and H-CHCHOH protonation steps are shown as insets. The OCH-

HCH protonation to form $OCH_2CH^*$ was not considered in **b** according to **Supplementary Table 8**. This figure clearly demonstrates that OCHCH* exclusively leads to intermediates only accounting for $C_2$ Oxy formation, i.e., $OCHCH_2^*$ and $CH_2CHOH(l)$. $\Delta G_a$ present in the OCHCH reduction pathway are lower than that of CHC-HO protonation at reducing conditions; thus, the steps later than OCHCH formation should not limit the OCHCH pathway.

Besides $OCHCH_2^*$, $OCH_2CH^*$ and CHCHOH* are two possible intermediates through reduction of OCHCH* (**Supplementary Figure 8a**). The former, however, is almost 1.2 eV less stable than $OCHCH_2^*$ and direct OCH-HCH protonation seems to be unlikely (**Supplementary Table 8**). Adding hydrogen to the oxygen end of OCHCH* is shown to be kinetically facile, whereas subsequent reduction steps of CHCHOH* towards either $CH_2CHOH^*$ or CHCH* + $H_2O$ exhibit sluggish kinetics and thus block the CHCHOH pathway at $U_{RHE} > -0.63$ V (**Supplementary Figure 8b**). In addition, although these steps become more favorable than OCHCH-H protonation at more negative potentials, $CH_2CHOH$ formation has a barrier at least 0.46 eV lower than the barrier for CHCHOH dehydroxylation. Thus, altering the dominant reduction pathway for OCHCH* from OCHCH-H protonation to sequential CHCHO-H and H-CHCHOH steps does not affect the products formed through the OCHCH pathway.

*2.2.4. The $CH_2CO$ pathway*

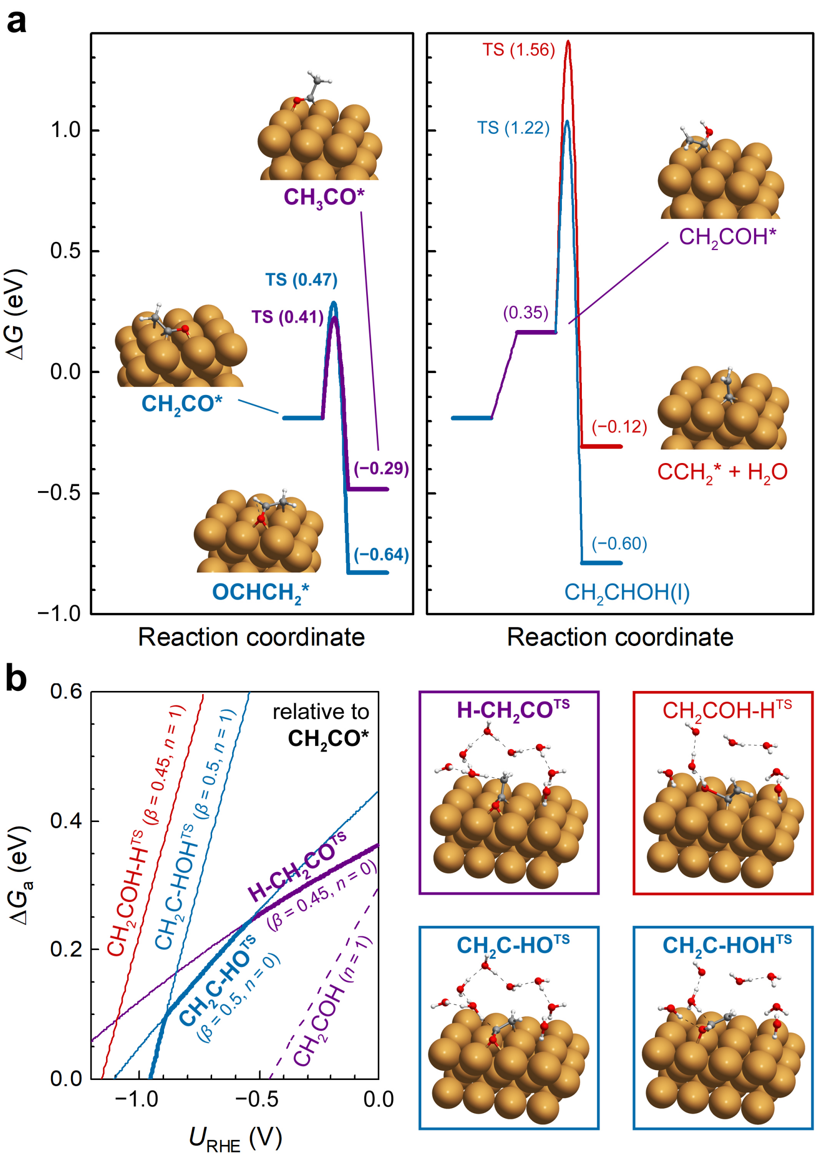


**Supplementary Figure 9. Energetic of $CH_2CO$ protonation on Cu(100) at pH7.** (**a**) FEDs at $U_{RHE}$ = 0 V. The protonation steps of α-C or the carbonyl bonds to form $CH_3CO$* (purple, bold), $OCHCH_2$* (blue), or $CH_2COH$* (purple) were considered. Further reduction steps of $CH_2COH$* to $CCH_2$* + $H_2O$ (red) or $CH_2CHOH$(l) (blue) were also considered as the $CH_2COH$ formation from OCHCH* is shown to be kinetically facile. Solvation corrections were applied and all the energies were referenced to CO(g), $H_2$(g), and $H_2O$(g) according to the CHE model. Numbers in the parentheses represent the $\Delta G_{rxn}$ or $\Delta G_a$ relative to $CH_2CO$*. To simply the diagrams, field corrections were not applied. The optimized geometries of adsorbates on Cu(100) are shown as insets. (**b**) $U$-dependent $\Delta G_a$ of several protonation steps for $CH_2CO$* and $CH_2COH$*. The differences in $\beta$ and $n$ (the number of electron and proton transferred to $CH_2CO$* before the corresponding TS) and dependency on interfacial field were explicitly considered. $\Delta G_a$ was relative to $CH_2CO$*. TS structures of H-$CH_2CO$, $CH_2C$-HO, $CH_2COH$-H, and $CH_2C$-HOH protonation steps are shown as insets. $CH_2CO$ desorption and subsequent hydration to form acetic

acid, as shown in **Figure 2e**, was not considered in **b** to focus on only reduction steps. This figure clearly demonstrates that $CH_2CO^*$ exclusively leads to intermediates such as $CH_3CO^*$, $OCHCH_2^*$, and $CH_2CHOH(l)$. These intermediates only account for $C_2$ Oxy production according to this figure and **Supplementary Figure 10**. $\Delta G_a$ present in the $CH_2CO$ reduction pathway are lower than that of OCCH-H protonation at reducing conditions; thus, the steps later than $CH_2CO$ formation should not limit the $CH_2CO$ pathway. $CH_2C$-HO protonation exhibits the lowest $\Delta G_a$ in a $U_{RHE}$ range of −0.54 V to −0.89 V, and therefore was selected for comparison with $CH_2CO$ hydration in **Figure 2e** at $U_{RHE} = -0.73$ V.

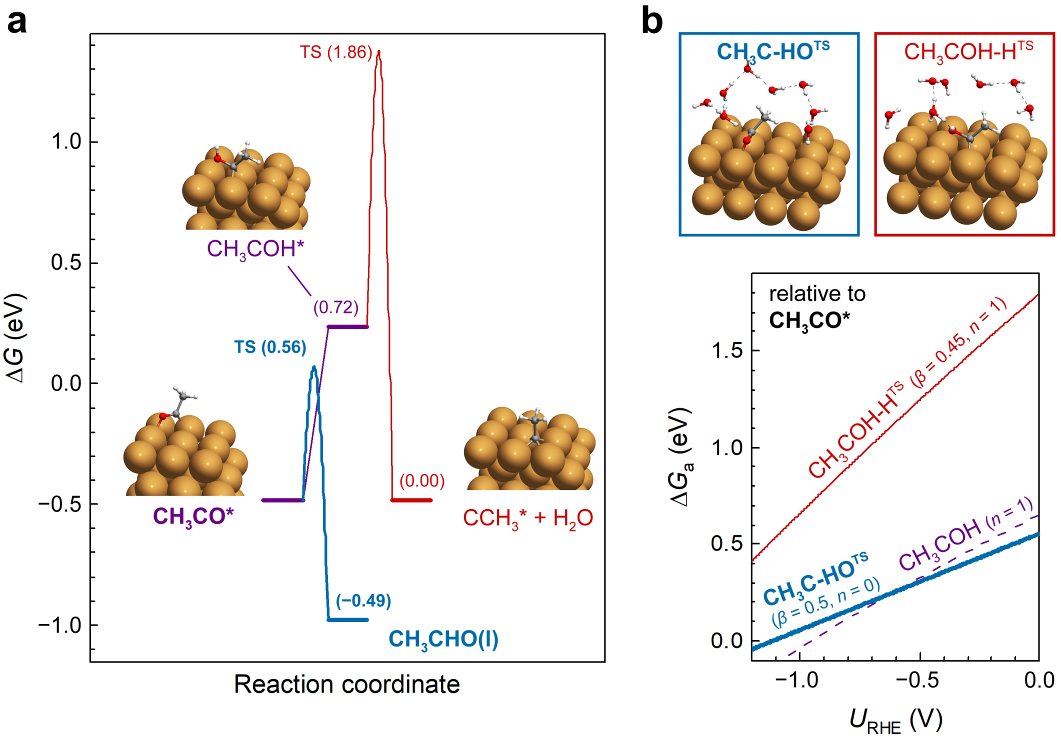


**Supplementary Figure 10. Energetic of $CH_3CO$ protonation on Cu(100) at pH7.** (**a**) FEDs at $U_{RHE} = 0$ V. The protonation steps of the carbonyl bonds to form $CH_3CHO(l)$ (blue) or $CH_3COH^*$ (purple) were considered. Further reduction steps of $CH_3COH^*$ to $CCH_3^* + H_2O$ (red) was also considered as the $CH_3COH$ formation from $CH_3CO^*$ is shown to be kinetically facile. Solvation corrections were applied and all the energies were referenced to CO(g), $H_2$(g), and $H_2O$(g) according to the CHE model. Numbers in the parentheses represent the $\Delta G_{rxn}$ or $\Delta G_a$ relative to $CH_3CO^*$. To simply the diagrams, field corrections were not applied. The optimized geometries of adsorbates on Cu(100) are shown as insets. (**b**) $U$-dependent $\Delta G_a$ of several protonation steps for $CH_3CO^*$ and $CH_3COH^*$. The differences in $\beta$ and $n$ (the number of electron and proton transferred to $CH_3CO^*$ before the corresponding TS) and dependency on interfacial field were explicitly considered. $\Delta G_a$ was relative to $CH_3CO^*$. TS structures of $CH_3C$-HO and $CH_3COH$-H protonation steps are shown as insets. This figure clearly demonstrates that $CH_3CO^*$ exclusively leads to $CH_3CHO$ (l). These intermediates only account for $C_2$ Oxy production according to this figure and **Supplementary Figure 9**. $\Delta G_a$ present in the $CH_3CO$ reduction pathway are lower than that of OCCH-H protonation at reducing conditions; thus, the steps later than $CH_2CO$ formation should not limit the $CH_2CO$ pathway.

Protonation of $CH_2CO^*$ is more complex than the ethenone hydration, resulting in surface species $CH_3CO^*$, $OCHCH_2^*$, and $CH_2COH^*$, respectively (**Supplementary Figure 9**). All

three steps are shown to be facile with a $\Delta G_a$ lower than 0.47 eV at $U_{RHE}$ = 0 V. Multiple reduction pathways for $CH_3CO$* and $CH_2COH$* were also considered (**Supplementary Figure 9** and **10**). Similar to the case of CHCHOH reduction and despite the kinetically facile protonation of carbonyl O to form a hydroxyl group, further dehydroxylation of $CH_2COH$* and $CH_3COH$* to $C_2$ HC intermediates is shown to be less favorable than the formation of either vinyl alcohol or acetaldehyde (**Supplementary Figure 9** and **10**). Therefore, similar as for the OCHCH pathway, the $CH_2CO$ pathway also exclusively leads to $C_2$ Oxys. The only difference lies in the ability of $CH_2CO$ pathway to produce acetate. The major competing reduction steps with the ethenone hydration are the protonation steps H-$CH_2CO$ and $CH_2C$-HO, of which the former dominates at lower overpotential ($U_{RHE} > -0.54$ V) whereas the latter dominates at higher overpotential ($U_{RHE} < -0.54$ V) (**Supplementary Figure 9b** and **Figure 2e**). Nevertheless, the difference in their $\Delta G_a$ is very small and both barriers are lower than that of OCCH-H protonation. Thus, these later steps play a negligible role in determining the $C_2$ Oxy/HC selectivity.

## 2.3. Microkinetic model of COR and pH effects

To prevent the interference from the different electron-transfer number per product molecule ($N$), as well as the difference in $N$ brought by the initial reactant of $CO_2$ and CO, we intentionally convert all the specific current densities of $CO_2R$ ($j_{CO2R}$) to current densities by assuming CO as the initial reactant ($j_{COR}$). Therefore, the $j_{CO2R}$ data reported in Ref.[51] could be directly compared with $j_{COR}$ data in Ref.[33] through the equation $j_{COR} = j_{CO2R} \times (N_{COR}/N_{CO2R})$ using the $N$ shown in **Supplementary Table 9**. For instance, the partial current density of $CH_4$ obtained from $CO_2R$ was converted to that obtained from COR by multiplying a factor of 6/8 = 3/4.

Besides, for simplicity and to prevent the issue of our microkinetic models to simulate the reduction rate of desorbed intermediate molecules to their most reduced forms (*e.g.* acetaldehyde to ethanol, propionaldehyde/allyl alcohol to *n*-propanol), we assume that these desorbed intermediate molecules could be eventually fully reduced and integrated the formation rate of these molecules to their most reduced forms as

- $j_{ethanol*} = j_{ethanol} + j_{acetaldehyde} \times (8/6)$, where 8 and 6 are the $N_{COR}$ for ethanol and acetaldehyde, respectively
- $j_{C3*} = j_{propanol} + j_{allyl\ alcohol} \times (12/10) + j_{propionaldehyde} \times (12/10)$, where 12 and 10 are the $N_{COR}$ for propanol and allyl alcohol/propionaldehyde, respectively

**Supplementary Table 9. Conversion of experimental $j_{CO2R}$ to $j_{COR}$ for fair comparison.** The data was used to plot **Figure 3b**.

| Product | Reactant | Reaction | $N$ | |
|---|---|---|---|---|
| | | | $CO_2R$ | COR |
| $CH_4$ | $CO_2$ | $CO_2 + 8(H^+ + e^-) \rightarrow CH_4 + 2H_2O$ | 8 | 6 |
| (*methane*) | CO | $CO + 6(H^+ + e^-) \rightarrow CH_4 + H_2O$ | | |
| $C_2H_4$ | $CO_2$ | $2CO_2 + 12(H^+ + e^-) \rightarrow C_2H_4 + 4H_2O$ | 12 | 8 |
| (*ethylene*) | CO | $2CO + 8(H^+ + e^-) \rightarrow C_2H_4 + 2H_2O$ | | |
| $C_2H_6$ | $CO_2$ | $2CO_2 + 14(H^+ + e^-) \rightarrow C_2H_6 + 4H_2O$ | 14 | 10 |
| (*ethane*) | CO | $2CO + 10(H^+ + e^-) \rightarrow C_2H_6 + 2H_2O$ | | |
| $CH_3COOH$ | $CO_2$ | $2CO_2 + 8(H^+ + e^-) \rightarrow CH_3COOH + 2H_2O$ | 8 | 4 |
| (*acetic acid*) | CO | $2CO + 4(H^+ + e^-) \rightarrow CH_3COOH$ | | |
| $CH_3CHO$ | $CO_2$ | $2CO_2 + 10(H^+ + e^-) \rightarrow CH_3CHO + 3H_2O$ | 10 | 6 |
| (*acetaldehyde*) | CO | $2CO + 6(H^+ + e^-) \rightarrow CH_3CHO + H_2O$ | | |
| $CH_3CH_2OH$ | $CO_2$ | $2CO_2 + 12(H^+ + e^-) \rightarrow CH_3CH_2OH + 3H_2O$ | 12 | 8 |
| (*ethanol*) | CO | $2CO + 8(H^+ + e^-) \rightarrow CH_3CH_2OH + H_2O$ | | |
| $CH_3CH_2CHO$ | $CO_2$ | $3CO_2 + 16(H^+ + e^-) \rightarrow CH_3CH_2CHO + 5H_2O$ | 16 | 10 |
| (*propionaldehyde*) | CO | $3CO + 10(H^+ + e^-) \rightarrow CH_3CH_2CHO + 2H_2O$ | | |
| $CH_2CHCH_2OH$ | $CO_2$ | $3CO_2 + 16(H^+ + e^-) \rightarrow CH_2CHCH_2OH + 5H_2O$ | 16 | 10 |
| (*allyl alcohol*) | CO | $3CO + 10(H^+ + e^-) \rightarrow CH_2CHCH_2OH + 2H_2O$ | | |
| $CH_3CH_2CH_2OH$ | $CO_2$ | $3CO_2 + 18(H^+ + e^-) \rightarrow CH_3CH_2CH_2OH + 5H_2O$ | 18 | 12 |
| (*n-propanol*) | CO | $3CO_2 + 12(H^+ + e^-) \rightarrow CH_3CH_2CH_2OH + 5H_2O$ | | |

**Supplementary Table 10. Tabulated product distribution of experimental $CO_2R$ (0.1 M $KHCO_3$, pH7) and COR (0.1M KOH, pH13) on various Cu catalysts.** Data points in strongly mass-transport-limited region were not considered for plotting **Figure 3c** and **Supplementary Figure 12**. All the $j_{COR}$ for $CO_2R$ were obtained through the approach described as above and in **Supplementary Table 9**. All the $j_{COR}$ for COR were obtained directly from the Faradiac yield data and total current density data in the corresponding reference.

**Planar pcCu electrode, 0.1 M $KHCO_3$, Ref.[51]**

| $U_{RHE}$ (V) | Partial current density $j_{COR}$ (mA cm$^{-2}$) | | | | | Molecular ratio | |
|---|---|---|---|---|---|---|---|
| | $CH_4$ | $C_2H_4$ | EtOH | $AcO^-$ | $C_3$ | $C_2$ Oxy/HC | EtOH/$C_2H_4$ |
| −0.96 | 0.043 | 0.130 | 0.034 | 0.0008 | 0.044 | 0.274 | 0.261 |
| −1.01 | 0.466 | 0.426 | 0.139 | 0.0044 | 0.153 | 0.346 | 0.326 |
| −1.05 | 1.082 | 1.023 | 0.400 | 0.0088 | 0.155 | 0.408 | 0.391 |
| −1.09 | 2.049 | 1.288 | 0.585 | 0.011 | 0.194 | 0.471 | 0.454 |
| −1.14 | 3.665 | 1.251 | 0.468 | 0.0095 | 0.087 | 0.390 | 0.374 |

**Planar ED-Cu electrode, 0.1 M $KHCO_3$, Ref.[52]**

| $U_{RHE}$ (V) | Partial current density $j_{COR}$ (mA cm$^{-2}$) | | | | | Molecular ratio | |
|---|---|---|---|---|---|---|---|
| | $CH_4$ | $C_2H_4$ | EtOH | $AcO^-$ | $C_3$ | $C_2$ Oxy/HC | EtOH/$C_2H_4$ |
| −0.90 | 0.0056 | 0.013 | 0.0052 | - | 0.013 | 0.407 | 0.407 |
| −0.95 | 0.029 | 0.075 | 0.039 | 0.0059 | 0.061 | 0.674 | 0.516 |
| −1.00 | 0.240 | 0.363 | 0.148 | 0.016 | 0.169 | 0.498 | 0.408 |
| −1.05 | 1.986 | 1.903 | 0.868 | 0.063 | 0.503 | 0.522 | 0.456 |
| −1.10 | 4.211 | 1.876 | 1.128 | 0.116 | 0.447 | 0.725 | 0.601 |
| −1.15 | 8.611 | 1.054 | 0.649 | 0.071 | 0.150 | 0.750 | 0.616 |
| −1.20 | 14.207 | 0.852 | 0.570 | 0.047 | 0.089 | 0.779 | 0.669 |
| −1.25 | 13.250 | 0.311 | 0.190 | 0.022 | 0.052 | 0.751 | 0.609 |
| −1.30 | 3.546 | 0.083 | 0.137 | - | 0.042 | 1.643 | 1.643 |

**OD-Cu electrode, 0.1 M $KHCO_3$, Ref.[52]**

| $U_{RHE}$ (V) | Partial current density $j_{COR}$ (mA cm$^{-2}$) | | | | | Molecular ratio | |
|---|---|---|---|---|---|---|---|
| | $CH_4$ | $C_2H_4$ | EtOH | $AcO^-$ | $C_3$ | $C_2$ Oxy/HC | EtOH/$C_2H_4$ |
| −0.90 | 0.019 | 0.297 | 0.109 | - | 0.166 | 0.368 | 0.368 |
| −0.95 | 0.120 | 0.814 | 0.250 | - | 0.334 | 0.307 | 0.307 |
| −1.00 | 0.569 | 1.672 | 0.555 | - | 0.523 | 0.332 | 0.332 |
| −1.05 | 2.758 | 4.041 | 1.541 | 0.041 | 0.728 | 0.402 | 0.381 |
| −1.10 | 6.183 | 4.391 | 1.944 | 0.041 | 0.593 | 0.462 | 0.443 |
| −1.15 | 9.810 | 2.177 | 1.092 | 0.012 | 0.221 | 0.512 | 0.502 |
| −1.20 | 8.071 | 0.602 | 0.419 | - | 0.070 | 0.696 | 0.696 |

**Epitaxial grown single-crystal Cu(111) electrode, 0.1 M $KHCO_3$, Ref.[53]**

| $U_{RHE}$ (V) | Partial current density $j_{COR}$ (mA cm$^{-2}$) | | | | | Molecular ratio | |
|---|---|---|---|---|---|---|---|
| | $CH_4$ | $C_2H_4$ | EtOH | $AcO^-$ | $C_3$ | $C_2$ Oxy/HC | EtOH/$C_2H_4$ |
| −0.98 | 0.164 | 0.375 | 0.097 | 0.0081 | 0.112 | 0.302 | 0.259 |
| −1.04 | 1.589 | 0.865 | 0.412 | 0.015 | 0.218 | 0.512 | 0.476 |
| −1.10 | 3.534 | 1.158 | 0.590 | 0.017 | 0.145 | 0.538 | 0.509 |

**Epitaxial grown single-crystal Cu(100) electrode, 0.1 M $KHCO_3$, Ref.[53]**

| $U_{RHE}$ (V) | Partial current density $j_{COR}$ (mA cm$^{-2}$) | | | | | Molecular ratio | |
|---|---|---|---|---|---|---|---|
| | $CH_4$ | $C_2H_4$ | EtOH | $AcO^-$ | $C_3$ | $C_2$ Oxy/HC | EtOH/$C_2H_4$ |
| −0.89 | 0.020 | 0.172 | 0.014 | 0.0021 | 0.024 | 0.108 | 0.083 |

| | | | | | | | |
|---|---|---|---|---|---|---|---|
| −0.98 | 0.146 | 0.598 | 0.228 | 0.0058 | 0.150 | 0.400 | 0.381 |
| −1.04 | 1.056 | 1.437 | 0.497 | 0.012 | 0.204 | 0.363 | 0.346 |
| −1.10 | 3.701 | 1.210 | 0.431 | 0.011 | 0.059 | 0.374 | 0.356 |

**Epitaxial grown single-crystal Cu(751) electrode, 0.1 M $KHCO_3$, Ref.[53]**

| $U_{RHE}$ (V) | Partial current density $j_{COR}$ (mA cm$^{-2}$) | | | | | Molecular ratio | |
|---|---|---|---|---|---|---|---|
| | $CH_4$ | $C_2H_4$ | EtOH | $AcO^-$ | $C_3$ | $C_2$ Oxy/HC | EtOH/$C_2H_4$ |
| −0.89 | 0.0064 | 0.072 | 0.020 | 0.0070 | 0.029 | 0.470 | 0.277 |
| −0.98 | 0.230 | 0.442 | 0.151 | 0.0058 | 0.142 | 0.368 | 0.342 |
| −1.04 | 1.075 | 1.133 | 0.533 | 0.0093 | 0.182 | 0.487 | 0.471 |
| −1.10 | 2.439 | 1.678 | 0.835 | 0.028 | 0.224 | 0.531 | 0.497 |

**Planar pcCu electrode, 0.1 M KOH, Ref.[33]**

| $U_{RHE}$ (V) | Partial current density $j_{COR}$ (mA cm$^{-2}$) | | | | | Molecular ratio | |
|---|---|---|---|---|---|---|---|
| | $CH_4$ | $C_2H_4$ | EtOH | $AcO^-$ | $C_3$ | $C_2$ Oxy/HC | EtOH/$C_2H_4$ |
| −0.44 | - | 0.0053 | 0.0054 | - | - | 1.021 | 1.021 |
| −0.49 | - | 0.030 | 0.0092 | 0.0082 | - | 0.841 | 0.303 |
| −0.53 | - | 0.105 | 0.040 | 0.016 | 0.050 | 0.682 | 0.376 |
| −0.58 | - | 0.229 | 0.097 | 0.019 | 0.062 | 0.588 | 0.421 |
| −0.63 | - | 0.400 | 0.173 | 0.030 | 0.075 | 0.584 | 0.432 |
| −0.68 | - | 0.491 | 0.215 | 0.026 | 0.049 | 0.543 | 0.439 |
| −0.73 | 0.014 | 0.596 | 0.216 | 0.032 | - | 0.468 | 0.362 |
| −0.78 | 0.163 | 0.472 | 0.221 | 0.050 | - | 0.680 | 0.468 |
| −0.80 | 0.947 | 0.101 | 0.051 | 0.026 | - | 1.012 | 0.500 |

**Planar pcCu electrode, 0.1 M KOH, Ref.[54]**

| $U_{RHE}$ (V) | Partial current density $j_{COR}$ (mA cm$^{-2}$) | | | | | Molecular ratio | |
|---|---|---|---|---|---|---|---|
| | $CH_4$ | $C_2H_4$ | EtOH | $AcO^-$ | $C_3$ | $C_2$ Oxy/HC | EtOH/$C_2H_4$ |
| −0.40 | - | 0.024 | 0.031 | - | 0.044 | 1.288 | 1.288 |
| −0.50 | - | 0.162 | 0.054 | 0.012 | 0.182 | 0.483 | 0.332 |
| −0.59 | - | 0.630 | 0.325 | 0.025 | 0.259 | 0.597 | 0.516 |

**Nanoparticle pcCu electrode, 0.1 M KOH, Ref.[55]**

| $U_{RHE}$ (V) | Partial current density $j_{COR}$ (mA cm$^{-2}$) | | | | | Molecular ratio | |
|---|---|---|---|---|---|---|---|
| | $CH_4$ | $C_2H_4$ | EtOH | $AcO^-$ | $C_3$ | $C_2$ Oxy/HC | EtOH/$C_2H_4$ |
| −0.40 | - | - | 0.011 | 0.0060 | - | - | - |
| −0.45 | - | 0.0046 | 0.034 | 0.0026 | - | 8.571 | 7.429 |
| −0.50 | - | 0.047 | 0.053 | 0.0078 | - | 1.458 | 1.125 |
| −0.60 | - | 0.075 | 0.024 | 0.0040 | 0.044 | 0.421 | 0.316 |

**OD-Cu 1 electrode, 0.1 M KOH, Ref.[55]**

| $U_{RHE}$ (V) | Partial current density $j_{COR}$ (mA cm$^{-2}$) | | | | | Molecular ratio | |
|---|---|---|---|---|---|---|---|
| | $CH_4$ | $C_2H_4$ | EtOH | $AcO^-$ | $C_3$ | $C_2$ Oxy/HC | EtOH/$C_2H_4$ |
| −0.25 | - | - | 0.030 | 0.017 | - | - | - |
| −0.30 | - | 0.0017 | 0.122 | 0.039 | - | 116.833 | 71.500 |
| −0.35 | - | 0.0075 | 0.224 | 0.043 | - | 41.167 | 29.833 |
| −0.40 | - | 0.051 | 0.339 | 0.046 | - | 8.380 | 6.608 |
| −0.45 | - | 0.095 | 0.391 | 0.041 | - | 4.978 | 4.109 |
| −0.50 | - | 0.171 | 0.388 | 0.033 | - | 2.655 | 2.265 |

**OD-Cu 2 electrode, 0.1 M KOH, Ref.[55]**

| $U_{RHE}$ (V) | Partial current density $j_{COR}$ (mA cm$^{-2}$) | | | | | Molecular ratio | |
|---|---|---|---|---|---|---|---|
| | $CH_4$ | $C_2H_4$ | EtOH | $AcO^-$ | $C_3$ | $C_2$ Oxy/HC | EtOH/$C_2H_4$ |
| −0.30 | - | - | 0.046 | 0.025 | - | - | - |
| −0.35 | - | 0.010 | 0.126 | 0.053 | - | 23.932 | 12.991 |
| −0.40 | - | 0.059 | 0.209 | 0.056 | 0.081 | 5.448 | 3.545 |
| −0.45 | - | 0.084 | 0.184 | 0.021 | 0.061 | 2.681 | 2.188 |
| −0.50 | - | 0.202 | 0.271 | 0.013 | 0.039 | 1.471 | 1.339 |

**OD-Cu on a gas diffusion layer (GDL), 0.1 M KOH, Ref.[46]**

| $U_{RHE}$ (V) | Partial current density $j_{COR}$ (mA cm$^{-2}$) | | | | | Molecular ratio | |
|---|---|---|---|---|---|---|---|
| | $CH_4$ | $C_2H_4$ | EtOH | $AcO^-$ | $C_3$ | $C_2$ Oxy/HC | EtOH/$C_2H_4$ |
| −0.33 | - | 0.058 | 0.236 | 0.084 | - | 6.966 | 4.069 |
| −0.43 | - | 0.490 | 0.495 | 0.250 | 0.780 | 2.031 | 1.010 |
| −0.51 | - | 2.505 | 1.035 | 0.240 | 1.980 | 0.605 | 0.413 |
| −0.57 | - | 7.385 | 2.520 | 0.595 | 3.780 | 0.502 | 0.341 |
| −0.66 | - | 20.880 | 8.100 | 2.070 | 8.820 | 0.586 | 0.388 |
| −0.73 | - | 30.510 | 22.680 | 6.075 | 14.985 | 1.142 | 0.743 |

**OD-Cu nanowire (NW), 0.1 M KOH, Ref.[56]**

| $U_{RHE}$ (V) | Partial current density $j_{COR}$ (mA cm$^{-2}$) | | | | | Molecular ratio | |
|---|---|---|---|---|---|---|---|
| | $CH_4$ | $C_2H_4$ | EtOH | $AcO^-$ | $C_3$ | $C_2$ Oxy/HC | EtOH/$C_2H_4$ |
| −0.25 | - | 0.00013 | 0.023 | 0.017 | - | 440.926 | 174.753 |
| −0.30 | - | 0.0023 | 0.110 | 0.032 | - | 75.469 | 47.692 |
| −0.35 | - | 0.013 | 0.139 | 0.031 | - | 16.043 | 11.036 |
| −0.40 | - | 0.030 | 0.083 | 0.010 | 0.0039 | 3.464 | 2.805 |
| −0.45 | - | 0.034 | 0.046 | 0.003 | 0.0065 | 1.525 | 1.338 |

**OD-Cu nanoflower (NF), 0.1 M KOH, Ref.[57]**

| $U_{RHE}$ (V) | Partial current density $j_{COR}$ (mA cm$^{-2}$) | | | | | Molecular ratio | |
|---|---|---|---|---|---|---|---|
| | $CH_4$ | $C_2H_4$ | EtOH | $AcO^-$ | $C_3$ | $C_2$ Oxy/HC | EtOH/$C_2H_4$ |
| −0.23 | - | 0.0008 | 0.198 | 0.050 | - | 364.085 | 242.239 |
| −0.28 | - | 0.0016 | 0.255 | 0.179 | - | 382.740 | 159.490 |
| −0.33 | - | 0.014 | 0.471 | 0.164 | 0.0051 | 57.511 | 33.916 |
| −0.38 | - | 0.057 | 0.548 | 0.148 | 0.0043 | 14.764 | 9.591 |
| −0.43 | - | 0.174 | 0.524 | 0.111 | 0.0080 | 4.280 | 3.004 |

Note: EtOH refers to the total production rate of ethanol and acetaldehyde; $AcO^-$ referes to acetate; $C_3$ refers to the total production rate of propanol by assuming all other $C_3$ products could be fully reduced to propanol. From the above treatment, the $j_{COR}$ could be directly used to assess the reaction rate in mol s$^{-1}$ cm$^{-2}$; all the $j$ is normalized to geometric electrode surface area.

A previous study by Ringe *et al.* has suggested that both the ion identity and the CO adsorption could affect the interfacial field effect on $CO_2R$.[18] Therefore, the selection of parameters in our electric field model, i.e., the zero-charge potential ($U_{PZC}$) and charge-separation distance $d$ (see **Supplementary Note 4** for more details) might have an effect on our results. To investigate that, we performed sensitivity analyses of both the key energies (TS energies of the three SDSs) and the $C_2$ Oxy/HC ratio by varying $U_{PZC}$ and $d$ within reasonable ranges. The value of $d$ varies from 1.2 Å to 2.0, 3.0, 4.0, 5.0, and 6.0 Å. The 4.0, 5.0, and 6.0 Å approximate the effective interfacial cation radius of $K^+$, $Na^+$, and $Li^+$, respectively. The value of $U_{PZC}$ varies from −0.54 $V_{SHE}$ (for Cu(100)), to −0.20 $V_{SHE}$ (for Cu(111)), to 0.09 $V_{SHE}$ (for pcCu). All these values were directly adopted from Ref.[18]. The influence of varying $U_{PZC}$ and $d$ is shown in **Supplementary Figure 11**. Although changing these parameters in our electric field model has an effect on the TS energies, the energetic difference, e.g., $\Delta G_{a,0}^{\text{CHCO-H}} - \Delta G_{a,0}^{\text{OCCH-H}}$, is hardly influenced (**Supplementary Figures 11a**, **c**). Thus the overall trend in $C_2$ Oxy/HC ratio is not altered (**Supplementary Figures 11b**, **d**). Note that the right leg in **Supplementary Figure 11d** suffer from larger deviations. This suggests a potential strategy to further engineer the $C_2$ Oxy/HC selectivity at low overpotentials via the $U_{PZC}$ effect.

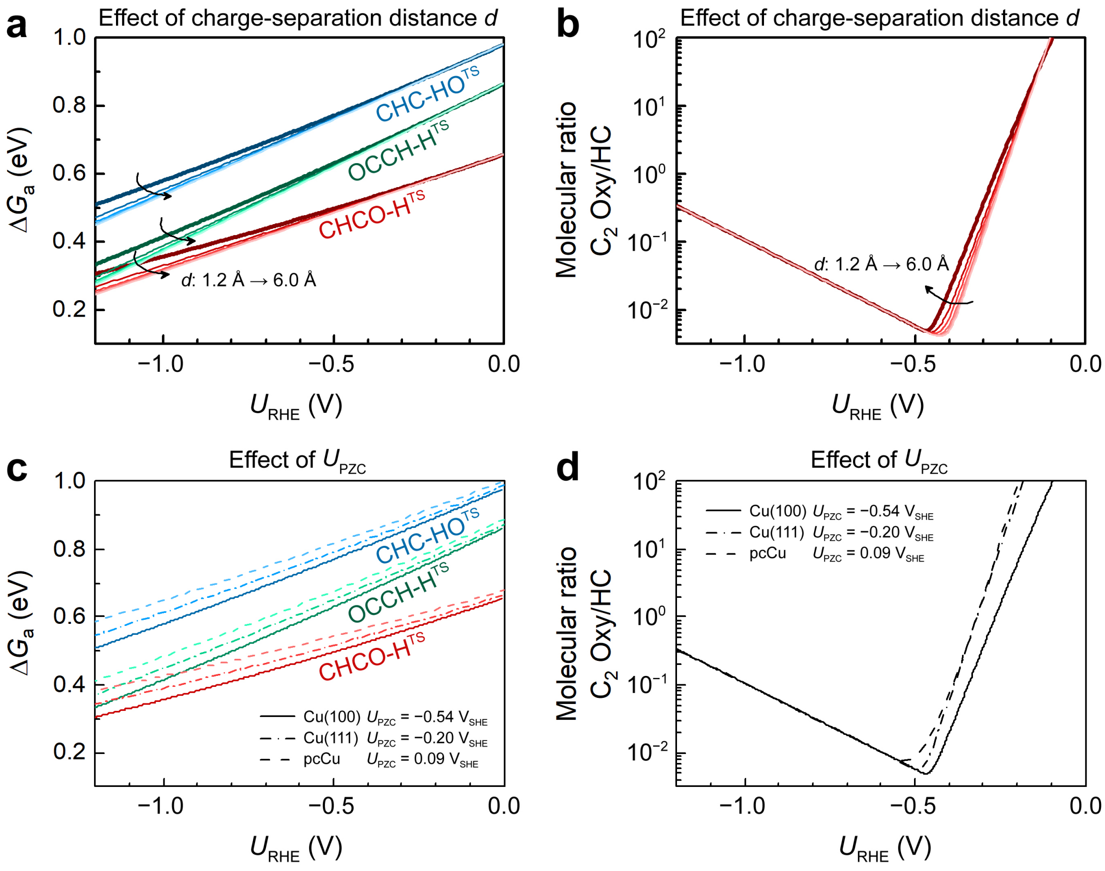


**Supplementary Figure 11. Sensitivity analyses with varying $U_{PZC}$ and *d*.** The effect of $d$ on (**a**) TS energies of CHCO-H, OCCH-H, and CHC-HO steps and (**b**) $C_2$ Oxy/HC ratio. The colors change from dark to light as $d$ increases. The bold lines refer to the case of $d$ = 1.2 Å, which is used throughout the other part of this work. For **a** and **b**, $U_{PZC}$ equals to −0.54 $V_{SHE}$ (for Cu(100)).

The effect of $U_{PZC}$ on (**c**) TS energies of CHCO-H, OCCH-H, and CHC-HO steps and (**d**) $C_2$ Oxy/HC ratio. The solid lines refer to the case of $U_{PZC}$ = −0.54 $V_{SHE}$, which is used throughout the other part of this work. For **c** and **d**, *d* equals to 1.2 Å.

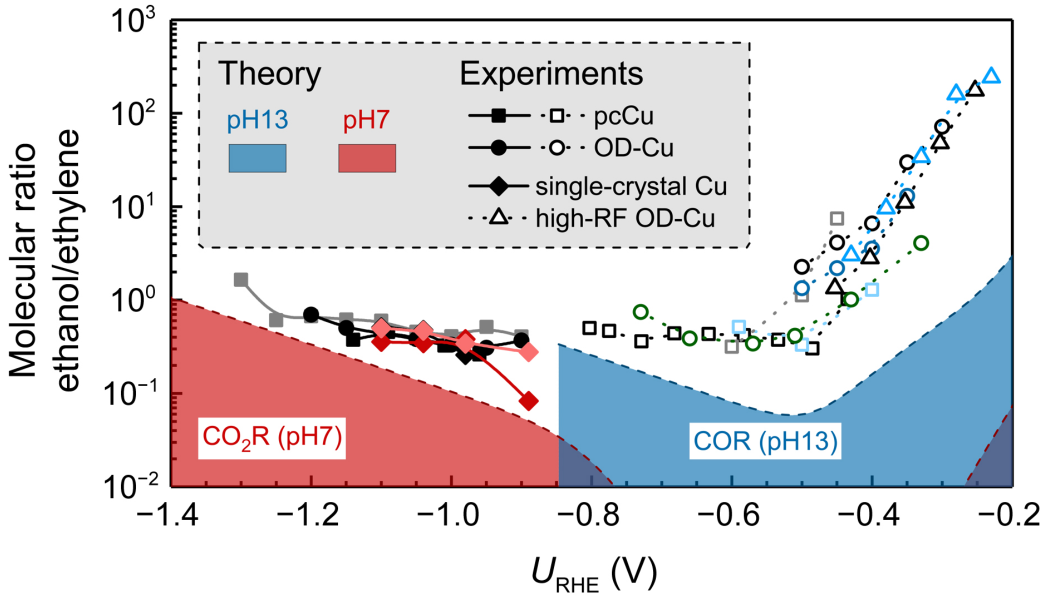


**Supplementary Figure 12. Trends in ethanol/ethylene ratio.** Ethanol/ethylene molecular ratios for $CO_2R$ (pH7) and COR (pH13) as a function of $U_{RHE}$ on a broad range of reported Cu-based catalysts in literatures. Solid and hollow plots correspond to data from $CO_2R$ (pH7) and COR (pH13), with solid and dotted lines to indicate the trend, respectively. Dashed lines in red and blue show the theoretically predicted trends in ethanol/ethylene molecular ratios at pH7 and pH13, respectively. The same works as shown in **Figure 3c** were referenced. All the data are listed in **Supplementary Table 10**.

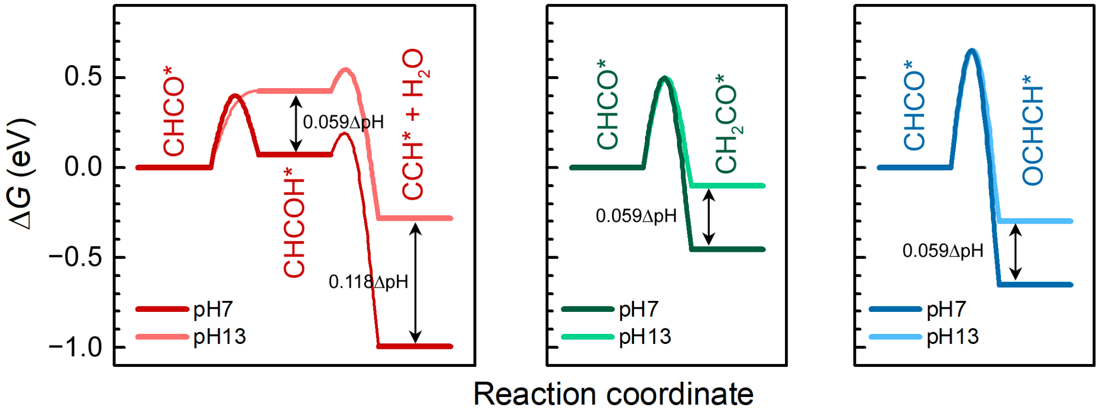


**Supplementary Figure 13. pH effects on the protonation steps of CHCO* at a constant absolute potential of $U_{SHE}$ = −1.14 V.** FEDs of three pathways: (*left*) the CHCOH pathway (CHCO* → CHCOH* → CCH* + $H_2O$* → … → $C_2$ HC); (*middle*) the $CH_2CO$ pathway (CHCO* → $CH_2CO$* → … → $C_2$ Oxy); (*right*) the OCHCH pathway (CHCO* → OCHCH* → … → $C_2$ Oxy). The pH13 represents either the pH of bulk solution as 13 or the local pH of 13 in bulk neutral-pH solution. The two pH conditions are equivalent at the same absolute potential. Owing to the thermodynamic instability of CHCOH* compared to $CH_2CO$* and OCHCH*, the CHCOH pathway undergoes a transition of the highest energy state from the TS of the first protonation (CHCO-$H^{TS}$ in red) to the TS of the second protonation (CHCOH-$H^{TS}$ in pink) with the increasing pH. This picture rationalizes how high pH suppress the $C_2$ HC pathway at low overpotentials.

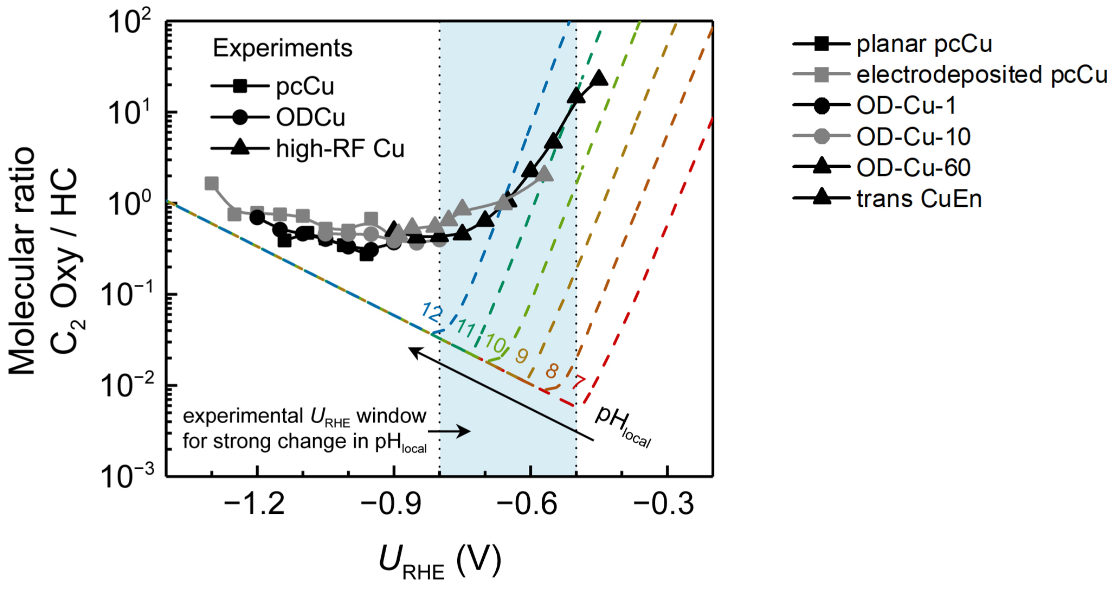


**Supplementary Figure 14. Trends in $C_2$ Oxy/HC ratio with local pH effect on $CO_2R$ in 0.1 M $KHCO_3$.** $C_2$ Oxy/HC molecular ratios for $CO_2R$ (pH7) with varying local pH. Solid plots correspond to data from $CO_2R$ (pH7); while dashed lines show the theoretically predicted trends at varying pH, respectively. Data from literatures: (low-RF Cu) planar pcCu;[51] electrodeposited pcCu and OD-Cu-1;[52] (high-RF Cu) OD-Cu-10 and OD-Cu-60;[52] in-situ transformed Cu nanoparticle ensemble, trans CuEn.[58] All the data are listed in **Table S11**.

**Supplementary Table 11. Tabulated product distribution of experimental $CO_2R$ (0.1 M $KHCO_3$, pH7) on various Cu catalysts.** Data points in strongly mass-transport-limited region were not considered for plotting. All the $j_{COR}$ for $CO_2R$ were obtained through the approach described as above and in **Supplementary Table 9**. All the $j_{COR}$ for COR were obtained directly from the Faradaic yield data and total current density data in the corresponding reference. Compared to the data present in **Supplementary Table 10**, additional results obtained on high-RF nanostructured electrodes are included as the local pH effect is normally more profound on high-RF electrode operated under larger geometric current densities.

**Planar pcCu electrode, 0.1 M $KHCO_3$, Ref.[51]**

| $U_{RHE}$ (V) | Partial current density $j_{COR}$ (mA $cm^{-2}$) | | | | | Molecular ratio | |
|---|---|---|---|---|---|---|---|
| | $CH_4$ | $C_2H_4$ | EtOH | $AcO^-$ | $C_3$ | $C_2$ Oxy/HC | EtOH/$C_2H_4$ |
| −0.96 | 0.043 | 0.130 | 0.034 | 0.0008 | 0.044 | 0.274 | 0.261 |
| −1.01 | 0.466 | 0.426 | 0.139 | 0.0044 | 0.153 | 0.346 | 0.326 |
| −1.05 | 1.082 | 1.023 | 0.400 | 0.0088 | 0.155 | 0.408 | 0.391 |
| −1.09 | 2.049 | 1.288 | 0.585 | 0.011 | 0.194 | 0.471 | 0.454 |
| −1.14 | 3.665 | 1.251 | 0.468 | 0.0095 | 0.087 | 0.390 | 0.374 |

**Planar ED-Cu electrode, 0.1 M $KHCO_3$, Ref.[52]**

| $U_{RHE}$ (V) | Partial current density $j_{COR}$ (mA $cm^{-2}$) | | | | | Molecular ratio | |
|---|---|---|---|---|---|---|---|
| | $CH_4$ | $C_2H_4$ | EtOH | $AcO^-$ | $C_3$ | $C_2$ Oxy/HC | EtOH/$C_2H_4$ |
| −0.90 | 0.0056 | 0.013 | 0.0052 | - | 0.013 | 0.407 | 0.407 |
| −0.95 | 0.029 | 0.075 | 0.039 | 0.0059 | 0.061 | 0.674 | 0.516 |
| −1.00 | 0.240 | 0.363 | 0.148 | 0.016 | 0.169 | 0.498 | 0.408 |
| −1.05 | 1.986 | 1.903 | 0.868 | 0.063 | 0.503 | 0.522 | 0.456 |

| | | | | | | | |
|---|---|---|---|---|---|---|---|
| −1.10 | 4.211 | 1.876 | 1.128 | 0.116 | 0.447 | 0.725 | 0.601 |
| −1.15 | 8.611 | 1.054 | 0.649 | 0.071 | 0.150 | 0.750 | 0.616 |
| −1.20 | 14.207 | 0.852 | 0.570 | 0.047 | 0.089 | 0.779 | 0.669 |
| −1.25 | 13.250 | 0.311 | 0.190 | 0.022 | 0.052 | 0.751 | 0.609 |
| −1.30 | 3.546 | 0.083 | 0.137 | - | 0.042 | 1.643 | 1.643 |

**OD-Cu-1 electrode, 0.1 M $KHCO_3$, Ref.**[52]

| $U_{RHE}$ (V) | Partial current density $j_{COR}$ (mA $cm^{-2}$) | | | | | Molecular ratio | |
|---|---|---|---|---|---|---|---|
| | $CH_4$ | $C_2H_4$ | EtOH | $AcO^-$ | $C_3$ | $C_2$ Oxy/HC | EtOH/$C_2H_4$ |
| −0.90 | 0.019 | 0.297 | 0.109 | - | 0.166 | 0.368 | 0.368 |
| −0.95 | 0.120 | 0.814 | 0.250 | - | 0.334 | 0.307 | 0.307 |
| −1.00 | 0.569 | 1.672 | 0.555 | - | 0.523 | 0.332 | 0.332 |
| −1.05 | 2.758 | 4.041 | 1.541 | 0.041 | 0.728 | 0.402 | 0.381 |
| −1.10 | 6.183 | 4.391 | 1.944 | 0.041 | 0.593 | 0.462 | 0.443 |
| −1.15 | 9.810 | 2.177 | 1.092 | 0.012 | 0.221 | 0.512 | 0.502 |
| −1.20 | 8.071 | 0.602 | 0.419 | - | 0.070 | 0.696 | 0.696 |

**OD-Cu-10 electrode, 0.1 M $KHCO_3$, Ref.**[52]

| $U_{RHE}$ (V) | Partial current density $j_{COR}$ (mA $cm^{-2}$) | | | | | Molecular ratio | |
|---|---|---|---|---|---|---|---|
| | $CH_4$ | $C_2H_4$ | EtOH | $AcO^-$ | $C_3$ | $C_2$ Oxy/HC | EtOH/$C_2H_4$ |
| −0.80 | 0.00074 | 1.031 | 0.345 | 0.032 | 0.484 | 0.397 | 0.335 |
| −0.85 | 0.0045 | 2.454 | 0.796 | 0.051 | 0.965 | 0.366 | 0.324 |
| −0.90 | 0.020 | 4.618 | 1.669 | 0.061 | 1.244 | 0.388 | 0.361 |
| −0.95 | 0.066 | 7.127 | 3.150 | 0.052 | 1.299 | 0.457 | 0.442 |
| −1.00 | 0.153 | 7.846 | 3.540 | 0.032 | 0.870 | 0.459 | 0.451 |
| −1.05 | 0.452 | 5.555 | 2.524 | 0.021 | 0.461 | 0.462 | 0.454 |

**OD-Cu-60 electrode, 0.1 M $KHCO_3$, Ref.**[52]

| $U_{RHE}$ (V) | Partial current density $j_{COR}$ (mA $cm^{-2}$) | | | | | Molecular ratio | |
|---|---|---|---|---|---|---|---|
| | $CH_4$ | $C_2H_4$ | EtOH | $AcO^-$ | $C_3$ | $C_2$ Oxy/HC | EtOH/$C_2H_4$ |
| −0.45 | - | 0.00058 | 0.010 | 0.0016 | 0.0026 | 22.667 | 17.167 |
| −0.50 | - | 0.0024 | 0.022 | 0.0061 | 0.0088 | 14.487 | 9.310 |
| −0.55 | - | 0.014 | 0.031 | 0.018 | 0.016 | 4.612 | 2.139 |
| −0.60 | - | 0.060 | 0.067 | 0.034 | 0.051 | 2.234 | 1.108 |
| −0.65 | - | 0.262 | 0.178 | 0.048 | 0.185 | 1.047 | 0.678 |
| −0.70 | 0.0018 | 0.700 | 0.321 | 0.063 | 0.399 | 0.639 | 0.458 |
| −0.75 | 0.0012 | 1.424 | 0.513 | 0.066 | 0.707 | 0.453 | 0.360 |
| −0.80 | 0.0017 | 3.083 | 1.171 | 0.085 | 1.131 | 0.435 | 0.380 |
| −0.85 | 0.0037 | 4.552 | 1.815 | 0.047 | 1.175 | 0.420 | 0.399 |
| −0.90 | 0.0055 | 6.750 | 3.292 | 0.026 | 0.884 | 0.495 | 0.488 |

**Transformed Cu nanoparticle ensemble electrode, 0.1 M $KHCO_3$, Ref.**[58]

| $U_{RHE}$ (V) | Partial current density $j_{COR}$ (mA $cm^{-2}$) | | | | | Molecular ratio | |
|---|---|---|---|---|---|---|---|
| | $CH_4$ | $C_2H_4$ | EtOH | $AcO^-$ | $C_3$ | $C_2$ Oxy/HC | EtOH/$C_2H_4$ |
| −0.57 | - | 0.019 | 0.028 | 0.0055 | 0.016 | 2.016 | 1.446 |
| −0.66 | - | 0.110 | 0.081 | 0.013 | 0.052 | 0.972 | 0.736 |
| −0.75 | - | 0.396 | 0.280 | 0.029 | 0.162 | 0.853 | 0.706 |
| −0.78 | 0.0059 | 1.048 | 0.584 | 0.047 | 0.341 | 0.647 | 0.558 |
| −0.81 | 0.038 | 2.331 | 1.166 | 0.058 | 0.567 | 0.549 | 0.500 |
| −0.86 | 0.444 | 4.530 | 2.307 | 0.061 | 0.553 | 0.536 | 0.509 |

| | | | | | | | |
|---|---|---|---|---|---|---|---|
| −0.89 | 2.244 | 5.719 | 2.519 | 0.043 | 0.524 | 0.455 | 0.441 |

Note: EtOH refers to the total production rate of ethanol and acetaldehyde; $AcO^-$ referes to acetate; $C_3$ refers to the total production rate of propanol by assuming all other $C_3$ products could be fully reduced to propanol. From the above treatment, the $j_{COR}$ could be directly used to assess the reaction rate in mol $s^{-1}$ $cm^{-2}$; all the $j$ is normalized to geometric electrode surface area.

## 2.4. Selectivity maps with $\Delta G_{C*}$ and $\Delta G_{OH*}$ as descriptors

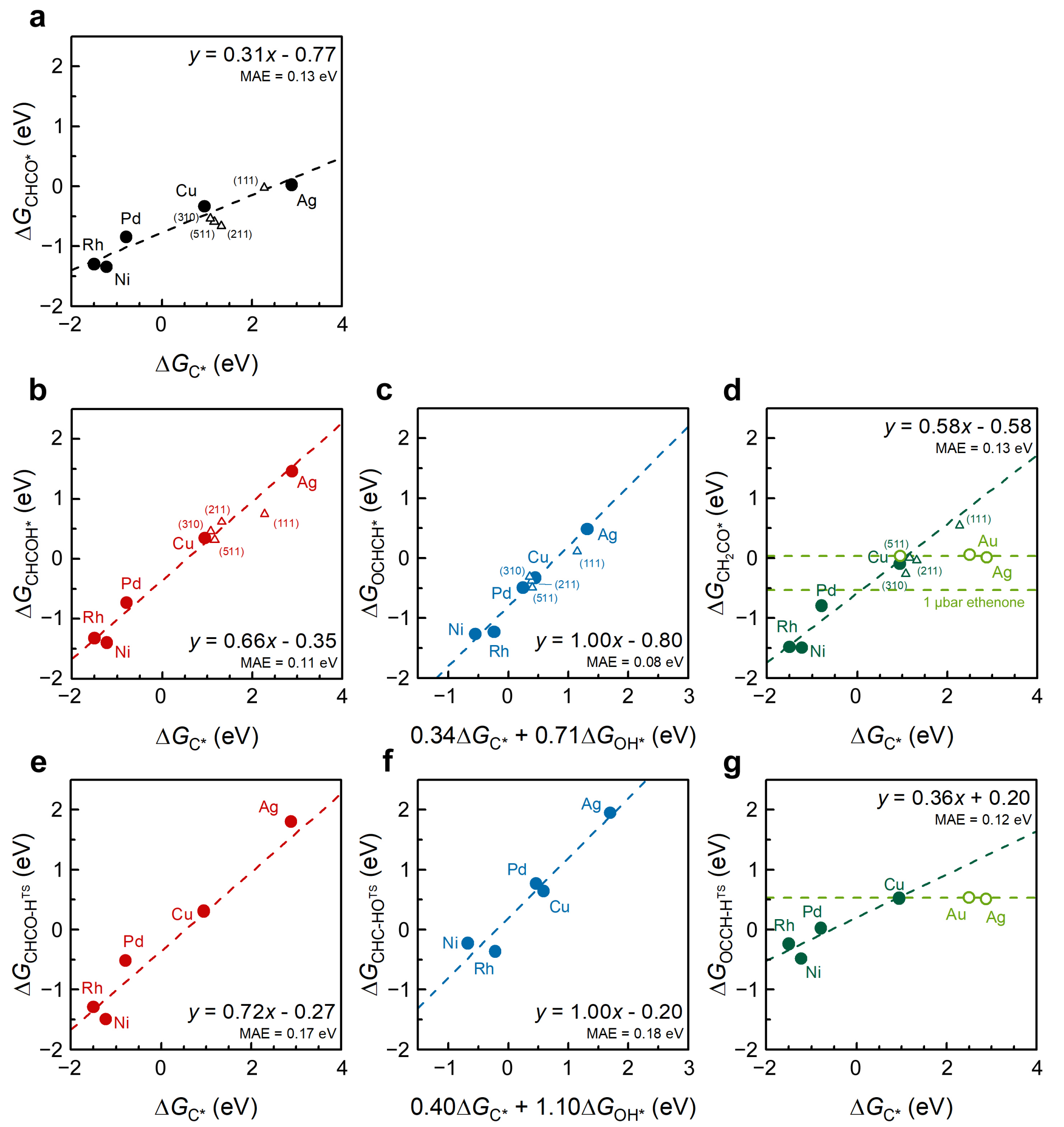


**Supplementary Figure 15. Energetic scaling lines of adsorption energies and TS energies on close-packed (100) metal surfaces with descriptors of $\Delta G_{C*}$ and $\Delta G_{OH*}$.** Adsorption energies of (**a**) $\Delta G_{CHCO*}$, (**b**) $\Delta G_{CHCOH*}$, (**c**) $\Delta G_{OCHCH*}$, and (**d**) $\Delta G_{CH2CO*}$. TS energies of (**e**) $\Delta G_{CHCO\text{-}H}$, (**f**) $\Delta G_{CHC\text{-}HO}$, and (**g**) $\Delta G_{OCCH\text{-}H}$. OCHCH* and CHC-HO$^{TS}$ were found to be described adequately by both $\Delta G_{C*}$ and $\Delta G_{OH*}$; whereas the other four energies only scale with $\Delta G_{C*}$ based on the bond-order analysis. Note that only the data presented by the solid circle, obtained on metal (100) surfaces, was used for parameterization. The 5*d* metals, Au and Pt, were excluded to construct the

(100) scaling lines because of the huge deviation using $\Delta G_{C*}$ on *5d* metal (100) surfaces.[5] Data obtained on other Cu facets than Cu(100) was also plotted as hollow triangles to show the trend in scaling relationships. For $CH_2CO$* and OCCH-$H^{TS}$, the data obtained on Ag was excluded for parameterization according to the bond-order conservation principle. To prove that, additional plots obtained on Au were provided in **d** and **g**. Noble metals such as Ag and Au are unable to cleave the $\pi$-bonds and thus the unsaturated double bonds in ethenone are preserved upon interaction with Ag and Au. The molecular ethenone tends to stay in the gas phase (light green dashed lines) rather than be chemisorbed on Ag and Au (hollow circles in green). Even on Cu(100), $CH_2CO$ chemisorbed on the surface is comparable in electronic energy with $CH_2CO$ in the vacuum layer. Therefore, the $CH_2CO$ and OCCH-HTS scaling relations tend to exhibit a slope of approximately 0 for noble metals. This concept has been demonstrated for unsaturated hydrocarbon species on elementary metals.[59] All the data are listed in **Supplementary Table 12**.

**Supplementary Table 12. Energetics of all species on various metals and intermetallics.** All adsorption/TS energies $\Delta G$ (in eV) are based on the formation reaction from reference gas-phase molecules of CO, $H_2O$, and $H_2$ (ref). TS energies were referenced to $U_{RHE}$ = 0V at pH7. Note that the solvation corrections are not applied herein. All computational data linked to the list of corresponding electronic energies has been released as part of the Catalysis-hub.org repository[6] under https://www.catalysis-hub.org/publications/PengTrends2022.

| *fcc*(100) | Ag | Au | Cu | Pd | Pt | Rh | Ni (spin) | Ni (non-spin) | Ir |
|---|---|---|---|---|---|---|---|---|---|
| C* | 2.87 | 2.50 | 0.95 | −0.80 | −0.56 | −1.24 | −1.18 | −1.51 | −1.12 |
| OH* | 0.49 | 1.27 | 0.18 | 0.71 | 1.20 | 0.25 | −0.08 | −0.06 | 0.70 |
| CHCO* | 0.03 | | −0.33 | −0.84 | | −1.33 | | −1.29 | |
| $CH_2CO$* | | | −0.09 | −0.79 | | −1.48 | | −1.47 | |
| $CH_2CO$ (desorbed) | 0.03 | 0.06 | 0.04 | | | | | | |
| OCHCH* | 0.49 | | −0.32 | −0.48 | | −1.23 | | −1.26 | |
| CHCOH* | 1.47 | | 0.36 | −0.72 | | −1.39 | | −1.31 | |
| OCCH-$H^{TS}$ | 0.53 | 0.54 | 0.53 | 0.03 | | −0.47 | | −0.23 | |
| CHC-$HO^{TS}$ | 1.96 | | 0.65 | 0.77 | | −0.36 | | −0.22 | |
| CHCO-$H^{TS}$ | 1.81 | | 0.32 | −0.51 | | −1.48 | | −1.28 | |
| Various Cu facets | (111) | (211) | (511) | (310) | (110) | | | | |
| C* | 2.28 | 1.32 | 1.16 | 1.36 | 1.57 | | | | |
| OH* | 0.54 | −0.08 | 0.01 | −0.01 | −0.22 | | | | |
| Alloys | $Cu_3Ag$ | $Cu_3Zn$ | CuAg | CuZn | | | | $Ni_5Ga_3$ (111) | |
| C* | 1.54 | 1.47 | 1.30 | 1.40 | | | | 0.39 | |
| OH* | 0.45 | 0.22 | 0.38 | 0.20 | | | | −0.10 | |

Note: Non-spin Ni was used for scaling parameterization as the CI-NEB calculations of spin Ni tend to be unconverged.

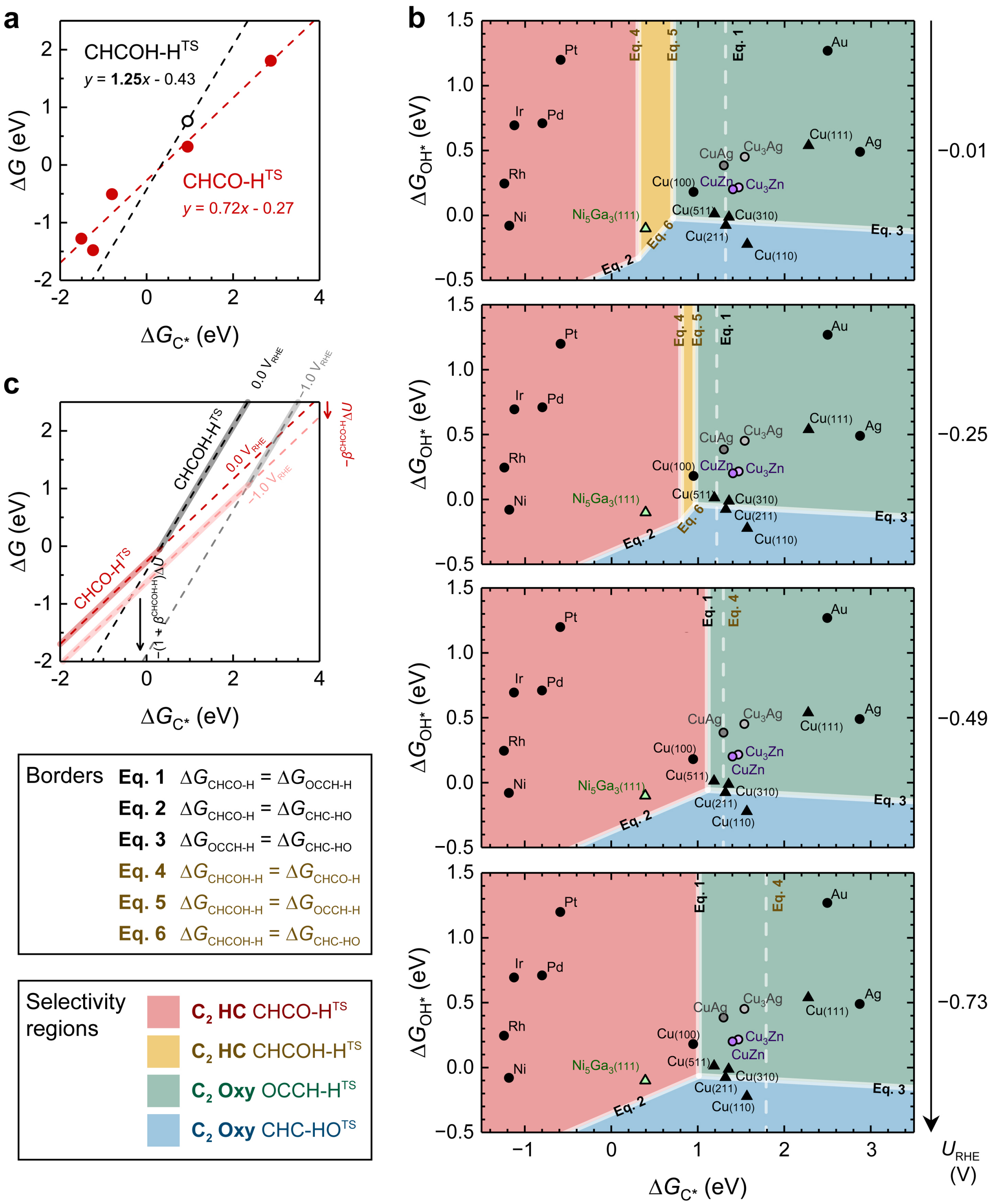


**Supplementary Figure 16. Potential dependency of the ($\Delta G_{C^*}$, $\Delta G_{OH^*}$) selectivity map.** (**a**) TS energies of $\Delta G_{\mathrm{CHCO\text{-}H}}$ and $\Delta G_{\mathrm{CHCOH\text{-}H}}$ with $\Delta G_{C^*}$ as the descriptor. The scaling relationship for $\Delta G_{\mathrm{CHCO\text{-}H}}$ was obtained using data from (100) surfaces of Ag, Cu, Pd, Ni, and Rh. The scaling relationship for $\Delta G_{\mathrm{CHCOH\text{-}H}}$ was constructed by assuming the slope to be 1.25, the same as for $\Delta G_{\mathrm{CCH^*}}$, according to the bond-order conservation assumption proposed by Jones *et al.*[59] (**b**) The ($\Delta G_{C^*}$, $\Delta G_{OH^*}$) selectivity maps with varying potentials, $U_{\mathrm{RHE}}$, from −0.01 to −0.73 V at pH7. The borders are defined by the equations shown in the left bottom, and the selectivity regions with corresponding dominant $C_2$ species and SDS were shown using different colors. (**c**) Scaling

relationships of $\Delta G_{\mathrm{CHCO\text{-}H}}$ and $\Delta G_{\mathrm{CHCOH\text{-}H}}$ with $\Delta G_{\mathrm{C}*}$ as the descriptor under $U_{\mathrm{RHE}}$ = 0.0 V (dark) and $U_{\mathrm{RHE}}$ = −1.0 V (light), respectively. The magnitudes the two scaling lines shift by potential are different, inducing the potential-dependent position of the border, Eq. 4, in **b**. With more negative potential, the border, Eq. 4, shifts from lower $\Delta G_{\mathrm{C}*}$ to higher $\Delta G_{\mathrm{C}*}$. **c** serves as an example to show how the selectivity map varies with potential.

## 3. Supplementary References


1. Giannozzi, P. et al. (2009) Quantum espresso: A modular and open-source software project for quantum simulations of materials. *J. Phys-Condens. Mat.* **21**, 395502.
2. Bahn, S. R. & Jacobsen, K. W. (2002) An object-oriented scripting interface to a legacy electronic structure code. *Comput. Sci. Eng.* **4**, 56-66.
3. Wellendorff, J. et al. (2012) Density functionals for surface science: Exchange-correlation model development with bayesian error estimation. *Phys. Rev. B* **85**, 235149.
4. Monkhorst, H. J. & Pack, J. D. (1976) Special points for brillouin-zone integrations. *Phys. Rev. B* **13**, 5188-5192.
5. Peng, H. J. et al. (2021) The role of atomic carbon in directing electrochemical co(2) reduction to multicarbon products. *Energy Environ. Sci.* **14**, 473-482.
6. Winther, K. T. et al. (2019) Catalysis-hub.Org an open electronic structure database for surface reactions. *Sci. Data* **6**, 75.
7. Peterson, A. A., Abild-Pedersen, F., Studt, F., Rossmeisl, J. & Nørskov, J. K. (2010) How copper catalyzes the electroreduction of carbon dioxide into hydrocarbon fuels. *Energy Environ. Sci.* **3**, 1311-1315.
8. Hori, Y., Murata, A. & Takahashi, R. (1989) Formation of hydrocarbons in the electrochemical reduction of carbon-dioxide at a copper electrode in aqueous-solution. *J. Chem. Soc.-Faraday Trans. I* **85**, 2309-2326.
9. Nitopi, S. et al. (2019) Progress and perspectives of electrochemical co2 reduction on copper in aqueous electrolyte. *Chem. Rev.* **119**, 7610-7672.
10. Christensen, R., Hansen, H. A. & Vegge, T. (2015) Identifying systematic dft errors in catalytic reactions. *Catal. Sci. Technol.* **5**, 4946-4949.
11. Studt, F. et al. (2015) The mechanism of co and co2 hydrogenation to methanol over cu-based catalysts. *ChemCatChem* **7**, 1105-1111.
12. Liu, X. Y. et al. (2019) Ph effects on the electrochemical reduction of co(2) towards c2 products on stepped copper. *Nat. Commun.* **10**, 32.
13. Ludwig, T. et al. (2019) Solvent-adsorbate interactions and adsorbate-specific solvent structure in carbon dioxide reduction on a stepped cu surface. *J. Phys. Chem. C* **123**, 5999-6009.
14. Xiao, H., Cheng, T., Goddard, W. A. & Sundararaman, R. (2016) Mechanistic explanation of the ph dependence and onset potentials for hydrocarbon products from electrochemical reduction of co on cu (111). *J. Am. Chem. Soc.* **138**, 483-486.
15. Garza, A. J., Bell, A. T. & Head-Gordon, M. (2018) Mechanism of co2 reduction at copper surfaces: Pathways to c2 products. *ACS Catal.* **8**, 1490-1499.
16. Montoya, J. H., Shi, C., Chan, K. & Nørskov, J. K. (2015) Theoretical insights into a co dimerization mechanism in co2 electroreduction. *J. Phys. Chem. Lett.* **6**, 2032-2037.
17. Nørskov, J. K., Studt, F., Abild-Pedersen, F. & Bligaard, T. Fundamental concepts in heterogeneous catalysis doi: 10.1002/9781118892114. (2014).
18. Ringe, S. et al. (2019) Understanding cation effects in electrochemical co2 reduction. *Energy Environ. Sci.* **12**, 3001-3014.

19. Grahame, D. C. (1947) The electrical double layer and the theory of electrocapillarity. *Chem. Rev.* **41**, 441-501.
20. Gauthier, J. A. et al. (2019) Unified approach to implicit and explicit solvent simulations of electrochemical reaction energetics. *J. Chem. Theory Comput.* **15**, 6895-6906.
21. Trasatti, S. & Lust, E. Modern aspects of electrochemistry doi. (Springer, 2002).
22. Nørskov, J. K. et al. (2004) Origin of the overpotential for oxygen reduction at a fuel-cell cathode. *J. Phys. Chem. B* **108**, 17886-17892.
23. Henkelman, G., Uberuaga, B. P. & Jonsson, H. (2000) A climbing image nudged elastic band method for finding saddle points and minimum energy paths. *J. Chem. Phys.* **113**, 9901-9904.
24. Hammer, B. in GPAW 2013: Users and developers meeting doi: (Technical University of Denmark; 2013).
25. Chan, K. & Nørskov, J. K. (2015) Electrochemical barriers made simple. *J. Phys. Chem. Lett.* **6**, 2663-2668.
26. Chan, K. & Nørskov, J. K. (2016) Potential dependence of electrochemical barriers from ab initio calculations. *J. Phys. Chem. Lett.* **7**, 1686-1690.
27. Trasatti, S. (1986) The absolute electrode potential: An explanatory note (recommendations 1986). *Pure Appl. Chem.* **58**, 955-966.
28. Liu, X. Y. et al. (2017) Understanding trends in electrochemical carbon dioxide reduction rates. *Nat. Commun.* **8**, 15438.
29. Strmcnik, D. et al. (2013) Improving the hydrogen oxidation reaction rate by promotion of hydroxyl adsorption. *Nat. Chem.* **5**, 300-306.
30. Lamoureux, P. S., Singh, A. R. & Chan, K. R. (2019) Ph effects on hydrogen evolution and oxidation over pt(111): Insights from first-principles. *ACS Catal.* **9**, 6194-6201.
31. Markovic, N. M., Grgur, B. N. & Ross, P. N. (1997) Temperature-dependent hydrogen electrochemistry on platinum low-index single-crystal surfaces in acid solutions. *J. Phys. Chem. B* **101**, 5405-5413.
32. Schmidt, T. J., Ross, P. N. & Markovic, N. M. (2002) Temperature dependent surface electrochemistry on pt single crystals in alkaline electrolytes: Part 2. The hydrogen evolution/oxidation reaction. *J. Electroanal. Chem.* **524**, 252-260.
33. Wang, L. et al. (2018) Electrochemical carbon monoxide reduction on polycrystalline copper: Effects of potential, pressure, and ph on selectivity toward multicarbon and oxygenated products. *ACS Catal.* **8**, 7445-7454.
34. Nie, X. W., Luo, W. J., Janik, M. J. & Asthagiri, A. (2014) Reaction mechanisms of co2 electrochemical reduction on cu(111) determined with density functional theory. *J. Catal.* **312**, 108-122.
35. Cheng, T., Xiao, H. & Goddard, W. A. (2015) Free-energy barriers and reaction mechanisms for the electrochemical reduction of co on the cu(100) surface, including multiple layers of explicit solvent at ph 0. *J. Phys. Chem. Lett.* **6**, 4767-4773.
36. Medford, A. J. et al. (2015) Catmap: A software package for descriptor-based microkinetic mapping of catalytic trends. *Catal. Lett.* **145**, 794-807.
37. Bothe, E., Dessouki, A. M. & Schultefrohlinde, D. (1980) Rate and mechanism of the ketene hydrolysis in aqueous solution. *J. Phys. Chem.* **84**, 3270-3272.

38. Clark, E. L. et al. (2019) Explaining the incorporation of oxygen derived from solvent water into the oxygenated products of co reduction over cu. *J. Am. Chem. Soc.* **141**, 4191-4193.
39. Lausche, A. C. et al. (2013) On the effect of coverage-dependent adsorbate-adsorbate interactions for co methanation on transition metal surfaces. *J. Catal.* **307**, 275-282.
40. Yang, N. Y. et al. (2016) Intrinsic selectivity and structure sensitivity of rhodium catalysts for c2+ oxygenate production. *J. Am. Chem. Soc.* **138**, 3705-3714.
41. Li, J. et al. (2019) Constraining co coverage on copper promotes high-efficiency ethylene electroproduction. *Nat. Catal.* **2**, 1124-1131.
42. Campbell, C. T. (1994) Micro- and macro-kinetics: Their relationship in heterogeneous catalysis. *Top. Catal.* **1**, 353-366.
43. Campbell, C. T. (2017) The degree of rate control: A powerful tool for catalysis research. *ACS Catal.* **7**, 2770-2779.
44. Lv, J. J. et al. (2018) A highly porous copper electrocatalyst for carbon dioxide reduction. *Adv. Mater.* **30**, 1803111.
45. Yang, K. L., Kas, R. & Smith, W. A. (2019) In situ infrared spectroscopy reveals persistent alkalinity near electrode surfaces during co2 electroreduction. *J. Am. Chem. Soc.* **141**, 15891-15900.
46. Jouny, M., Luc, W. & Jiao, F. (2018) High-rate electroreduction of carbon monoxide to multi-carbon products. *Nat. Catal.* **1**, 748-755.
47. Singh, M. R., Goodpaster, J. D., Weber, A. Z., Head-Gordon, M. & Bell, A. T. (2017) Mechanistic insights into electrochemical reduction of co2 over ag using density functional theory and transport models. *Proc. Natl. Acad. Sci. U. S. A.* **114**, E8812-E8821.
48. Ringe, S. et al. (2020) Double layer charging driven carbon dioxide adsorption limits the rate of electrochemical carbon dioxide reduction on gold. *Nat. Commun.* **11**, 33.
49. Patel, A. M., Vijay, S., Kastlunger, G., Nørskov, J. K. & Chan, K. (2021) Generalizable trends in electrochemical protonation barriers. *J. Phys. Chem. Lett.* **12**, 5193-5200.
50. Li, J., Stenlid, J. H., Ludwig, T., Lamoureux, P. S. & Abild-Pedersen, F. (2021) Modeling potential-dependent electrochemical activation barriers: Revisiting the alkaline hydrogen evolution reaction. (2021) *J. Am. Chem. Soc.* **143**, 19341-19355.
51. Kuhl, K. P., Cave, E. R., Abram, D. N. & Jaramillo, T. F. (2012) New insights into the electrochemical reduction of carbon dioxide on metallic copper surfaces. *Energy Environ. Sci.* **5**, 7050-7059.
52. Ren, D., Fong, J. H. & Yeo, B. S. (2018) The effects of currents and potentials on the selectivities of copper toward carbon dioxide electroreduction. *Nat. Commun.* **9**, 925.
53. Hahn, C. et al. (2017) Engineering cu surfaces for the electrocatalytic conversion of co2: Controlling selectivity toward oxygenates and hydrocarbons. *Proc. Natl. Acad. Sci. U. S. A.* **114**, 5918-5923.
54. Bertheussen, E. et al. (2018) Electroreduction of co on polycrystalline copper at low overpotentials. *ACS Energy Lett.* **3**, 634-640.

55. Li, C. W., Ciston, J. & Kanan, M. W. (2014) Electroreduction of carbon monoxide to liquid fuel on oxide-derived nanocrystalline copper. *Nature* **508**, 504-507.
56. Raciti, D. et al. (2017) Low-overpotential electroreduction of carbon monoxide using copper nanowires. *ACS Catal.* **7**, 4467-4472.
57. Wang, L. et al. (2019) Electrochemically converting carbon monoxide to liquid fuels by directing selectivity with electrode surface area. *Nat. Catal.* **2**, 702-708.
58. Kim, D., Kley, C. S., Li, Y. F. & Yang, P. D. (2017) Copper nanoparticle ensembles for selective electroreduction of co2 to c2-c3 products. *Proc. Natl. Acad. Sci. U. S. A.* **114**, 10560-10565.
59. Jones, G., Studt, F., Abild-Pedersen, F., Nørskov, J. K. & Bligaard, T. (2011) Scaling relationships for adsorption energies of c2 hydrocarbons on transition metal surfaces. *Chem. Eng. Sci.* **66**, 6318-6323.
